# A Continuously Benchmarked and Crowdsourced Challenge for Rapid Development and Evaluation of Models to Predict COVID-19 Diagnosis and Hospitalization

**DOI:** 10.1001/jamanetworkopen.2021.24946

**Published:** 2021-10-11

**Authors:** Yao Yan, Thomas Schaffter, Timothy Bergquist, Thomas Yu, Justin Prosser, Zafer Aydin, Amhar Jabeer, Ivan Brugere, Jifan Gao, Guanhua Chen, Jason Causey, Yuxin Yao, Kevin Bryson, Dustin R. Long, Jeffrey G. Jarvik, Christoph I. Lee, Adam Wilcox, Justin Guinney, Sean Mooney

**Affiliations:** 1 Sage Bionetworks, Seattle, Washington; 2 Molecular Engineering and Sciences Institute, University of Washington, Seattle; 3 Department of Biomedical Informatics and Medical Education, University of Washington, Seattle; 4 Institute of Translational Health Sciences, University of Washington, Seattle; 5 Department of Computer Engineering, Faculty of Engineering, Abdullah Gul University, Kayseri, Turkey; 6 Department of Computer Science, University of Illinois at Chicago, Chicago; 7 Department of Biostatistics and Medical Informatics, University of Wisconsin–Madison, Madison; 8 Computer Science Department, College of Engineering and Computer Science, Arkansas State University, Jonesboro; 9 Arkansas AI-Campus, Center for No-Boundary Thinking, Arkansas State University, Jonesboro; 10 Department of Computer Science, University College London, London, United Kingdom; 11 Division of Critical Care Medicine, Department of Anesthesiology and Pain Medicine, University of Washington, Seattle; 12 The University of Washington Clinical Learning, Evidence And Research Center for Musculoskeletal Disorders, Seattle; 13 Department of Radiology, University of Washington School of Medicine, Seattle

## Abstract

**Importance:**

Machine learning could be used to predict the likelihood of diagnosis and severity of illness. Lack of COVID-19 patient data has hindered the data science community in developing models to aid in the response to the pandemic.

**Objectives:**

To describe the rapid development and evaluation of clinical algorithms to predict COVID-19 diagnosis and hospitalization using patient data by citizen scientists, provide an unbiased assessment of model performance, and benchmark model performance on subgroups.

**Design, Setting, and Participants:**

This diagnostic and prognostic study operated a continuous, crowdsourced challenge using a model-to-data approach to securely enable the use of regularly updated COVID-19 patient data from the University of Washington by participants from May 6 to December 23, 2020. A postchallenge analysis was conducted from December 24, 2020, to April 7, 2021, to assess the generalizability of models on the cumulative data set as well as subgroups stratified by age, sex, race, and time of COVID-19 test. By December 23, 2020, this challenge engaged 482 participants from 90 teams and 7 countries.

**Main Outcomes and Measures:**

Machine learning algorithms used patient data and output a score that represented the probability of patients receiving a positive COVID-19 test result or being hospitalized within 21 days after receiving a positive COVID-19 test result. Algorithms were evaluated using area under the receiver operating characteristic curve (AUROC) and area under the precision recall curve (AUPRC) scores. Ensemble models aggregating models from the top challenge teams were developed and evaluated.

**Results:**

In the analysis using the cumulative data set, the best performance for COVID-19 diagnosis prediction was an AUROC of 0.776 (95% CI, 0.775-0.777) and an AUPRC of 0.297, and for hospitalization prediction, an AUROC of 0.796 (95% CI, 0.794-0.798) and an AUPRC of 0.188. Analysis on top models submitting to the challenge showed consistently better model performance on the female group than the male group. Among all age groups, the best performance was obtained for the 25- to 49-year age group, and the worst performance was obtained for the group aged 17 years or younger.

**Conclusions and Relevance:**

In this diagnostic and prognostic study, models submitted by citizen scientists achieved high performance for the prediction of COVID-19 testing and hospitalization outcomes. Evaluation of challenge models on demographic subgroups and prospective data revealed performance discrepancies, providing insights into the potential bias and limitations in the models.

## Key Points

**Question:**

What can be learned from a crowdsourced challenge for the prediction of COVID-19 diagnosis and hospitalization?

**Findings:**

This diagnostic and prognostic study used a model-to-data approach to implement a continuous benchmarking challenge that has enabled 482 participants to join in the effort to use regularly updated COVID-19 patient data to build machine learning models for COVID-19 diagnosis and hospitalization prediction. Machine learning models showed high accuracy in COVID-19 outcome prediction, but analysis of subgroups and prospective data revealed limitations and bias in the models.

**Meaning:**

This study suggests that crowdsourced clinical algorithms can predict COVID-19 diagnosis and hospitalization, but evaluation of the submitted models using reserved data sets is necessary to avoid self-assessment traps.

## Teaser

This diagnostic and prognostic study describes the development and evaluation of clinical algorithms to predict COVID-19 diagnosis and hospitalization using patient data by citizen scientists, provides an assessment of model performance, and benchmarks model performance on subgroups.

## Introduction

First reported in December 2019, the novel coronavirus SARS-CoV-2 has caused a global pandemic, resulting in strained hospital capacity and the deaths of 558 000 patients in the US alone as of April 7, 2021.^1^ As cumulative case counts increase, patient-level health data become a viable and crucial resource for researchers to understand disease patterns and design evidence-based interventions against the disease.^2^ Machine learning approaches applied to COVID-19 patient electronic health record (EHR) data have shown value in outbreak prediction,^3,4^ early screening,^5,6^ contact tracing of infected patients,^7,8^ health outcome prediction to improve diagnosis and treatment,^9^ and prioritization of health care resources for patients who are at a higher risk for health complications.^10,11^

Patient data must be acknowledged as private and sensitive, and there are appropriate restrictions in place for the sharing of these data, for example, the restrictions enumerated in the US Health Insurance Portability and Accountability Act. These necessary restrictions hinder data accessibility for researchers, limiting their ability to develop models and to externally validate their models. In cases where researchers have access to patient health data, models developed by isolated teams with no objective evaluation oversight can lead to self-assessment bias and overfit models.^12^

To overcome these challenges, we provided a solution for lowering the accessibility barrier to private patient data, while maintaining privacy protections, by implementing the model-to-data framework, under which investigators can build models on but never have direct access to sensitive health data.^13^ Using this infrastructure, we organized a response to the COVID-19 pandemic by launching the COVID-19 EHR DREAM Challenge. The feasibility and utility of this approach was previously demonstrated in the Patient Mortality EHR DREAM Challenge, leading to the unbiased assessment of machine learning models applied to EHRs to predict patient mortality.^14,15^ In our COVID-19 Challenge, we asked participants to address 2 clinically pressing questions. Diagnostic Question 1 (Q1): Of patients who received a test for COVID-19, who will have positive test results? Prognostic Question 2 (Q2): Of patients who have positive test results for COVID-19 in an outpatient setting, who is at risk for hospitalization within 21 days? The questions were motivated by the need to triage patients prior to widespread diagnostic and treatment capabilities. We evaluated models’ performance and generalizability to patient subgroups stratified by age, sex, race, ethnicity, and time of COVID-19 test.

## Methods

### Data

**Figure 1.**
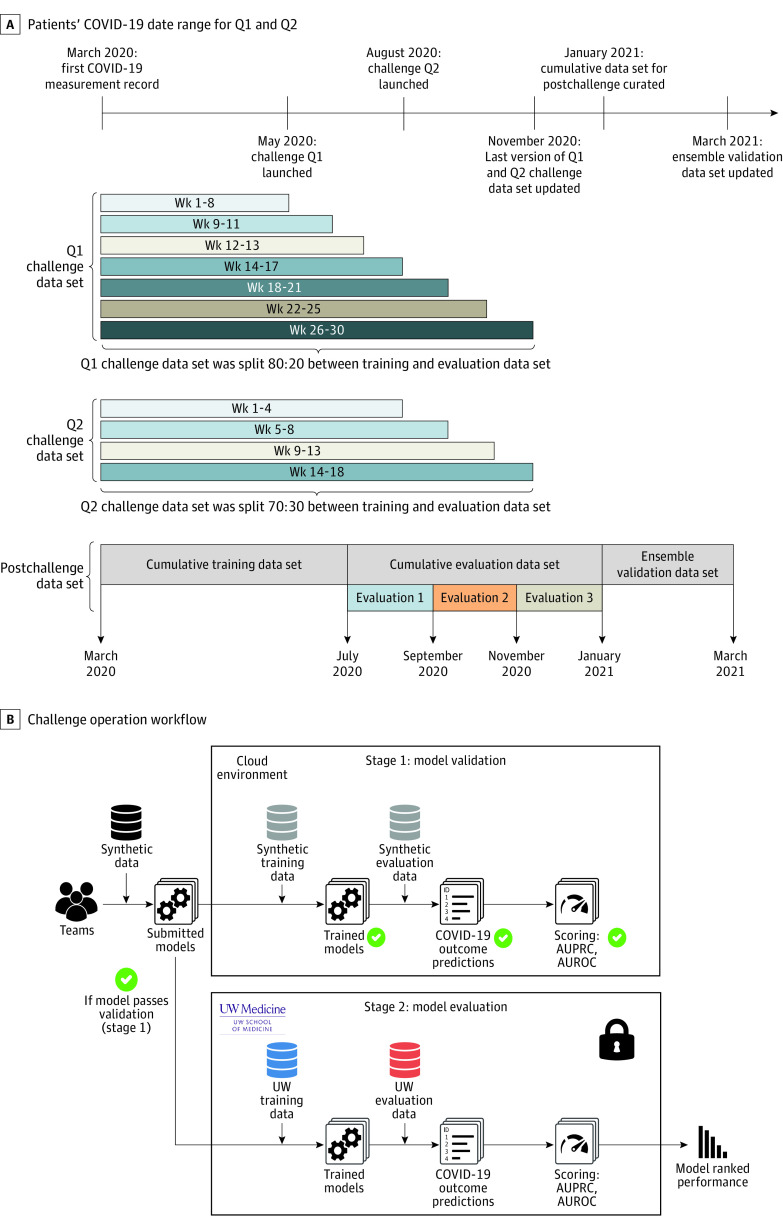
Visualization of the Challenge Timeline and Data A, Patients’ COVID-19 measurement date range in the data sets for question 1 (Q1) and question 2 (Q2). The plot includes both challenge data sets (used in the challenge) and cumulative data sets (used in postchallenge analysis). B, Challenge operation workflow. When a participant submitted a model to the challenge platform, Synapse, the model underwent a validation procedure on a cloud environment, in which the model was run against the synthetic data set (stage 1: model validation). If the model passed all the tests, the model was then pulled into a University of Washington (UW) secure environment, where it was trained and then applied to patient data (the holdout set from the full patient data set) to generate predictions (stage 2 model: evaluation). AUPRC indicates area under the precision recall curve; and AUROC, area under the receiver operating characteristic curve.

All work was reviewed and approved by the University of Washington (UW) institutional review board and UW Medicine leadership. We curated 2 challenge data sets (diagnostic Q1 challenge data set and prognostic Q2 challenge data set) separately for the purpose of model training and evaluation. The COVID-19 EHR DREAM challenge was run as a continuous benchmarking exercise where the data sets were updated every 2 to 5 weeks to incorporate new patients and update existing patients’ clinical trajectory. The Q1 challenge data set has 6 versions that accumulated over 30 weeks since May 6, 2020, and the Q2 challenge data set has 4 versions over 18 weeks since August 19, 2020. Each data set version was named by the challenge week during which the data set was used (Figure 1A; eAppendices 1 and 2 and eTables 1 and 2 in Supplement 1). This study followed the Transparent Reporting of a Multivariable Prediction Model for Individual Prognosis or Diagnosis (TRIPOD) reporting guideline.

In contrast to the last version of the Q1 and Q2 challenge data sets that were both updated November 18, 2020, we gathered all the data that had accumulated by January 21, 2021, and referred to this data set as the “cumulative data set.” This represented 108 500 patients who underwent testing for COVID-19, 4980 who received a positive test result, 3100 who received a positive test result during an outpatient visit, and 170 who were hospitalized within 21 days after receiving a positive test result during that outpatient visit. We split the cumulative data set in a way where 50% of patients who most recently underwent testing for COVID-19 were incorporated into the cumulative evaluation data set (patients who underwent testing between July 29, 2020, and January 21, 2021) and the other 50% were incorporated into the cumulative training data set (patients tested between March 2 and July 28, 2020). The cumulative evaluation data set was split evenly and prospectively into 3 subevaluation data sets based on the patients’ COVID-19 measurement date to evaluation 1 (July 29 to September 14, 2020), evaluation 2 (September 15 to November 11, 2020), and evaluation 3 (November 12, 2020, to January 21, 2021). (Figure 1A; eTable 3 in Supplement 1). The cumulative data set was used for postchallenge model analysis and training ensemble models.

We built an ensemble validation data set to evaluate the performance of ensemble models. This data set comprised 12 870 patients who had been tested for COVID-19 between January 22 and March 19, 2021, among which 278 had positive results, 208 had positive results in outpatient settings, and 16 were hospitalized within 21 days. (Figure 1A; eTable 3 in Supplement 1).

### Challenge Infrastructure and Workflow

We implemented the model-to-data approach for the COVID-19 challenge to facilitate the delivery of participants’ models to the sensitive challenge data sets. COVID-19 patient data sets were hosted on a UW Medicine Information Technology provisioned secure server. Challenge participants never had direct access to patient data; instead, they were required to build and submit Dockerized (containerized) models. A synthetic data set was provided to the participants to help them become familiar with the format of the data and to aid in technical debugging. Models submitted by participants would first go through a validation process in an Amazon Web Service cloud environment, running against synthetic data. Once validated, the models would be transferred to the UW environment, training and evaluating on real patient data. Area under the receiver operating characteristic curve (AUROC) and area under the precision recall curve (AUPRC) were 2 performance metrics we used to assess models. Synapse collaboration platform was used to receive submissions and host the challenge leaderboard (Figure 1B; for challenge computation resources, see eAppendix 3 in Supplement 1).

### Postchallenge Model Analysis

To evaluate and compare models submitted with different versions of challenge data sets, we retrained and evaluated Q1 and Q2 models separately on the cumulative data set. A full protocol of model retraining and selection is in eAppendix 4 in Supplement 1.

To evaluate and study the potential bias of the top 10 models from Q1 (eTable 4 in Supplement 1) on different strata of the patient population (eg, time of testing, age, sex, race, ethnicity), we trained these models on the cumulative training data set and evaluated their performance on subsets in the cumulative evaluation data set. For each stratum, we generated an AUROC score with a bootstrapped distribution (n = 1000; sample size = 10 000 with replacement). One-tailed *t* tests were used to examine if the top 10 models’ performances were consistently different and *P* < .001 was considered significant.

Valid submissions to Q2 from 7 independent teams were also retrained and evaluated on the cumulative training data set (eTable 5 in Supplement 1). The analysis for Q2 models focuses on 2 aspects: (1) if the model was used to predict 21-day hospitalization for all patients who had a positive COVID-19 test result regardless of the type of visit, would it be more or less accurate than predictions made for patients who were at an outpatient visit when they had a positive COVID-19 test result, and (2) if we limited the amount of patients’ pre–COVID-19 clinical history data available to model training, how would that be associated with a model’s performance? We generated AUROCs and bootstrapped distributions (n = 1000; sample size of 1000 with replacement) using 1-tailed *t* tests to assess performance differences.

### Ensemble Models

It has been shown that aggregating heterogeneous predictions from different models can improve individual model performance.^16,17^ We trained ensemble models for Q1 and Q2 separately using the top individual models (mentioned above). Trained on the cumulative training data set, each individual model outputs a probability between 0 and 1 indicating the likelihood of a patient receiving a positive COVID-19 test result (Q1) or being hospitalized within 21 days (Q2). A logistic regression model with 10-fold cross-validation ingested individual models’ probability for the cumulative evaluation data set to build an ensemble model. The ensemble validation data set was used to assess ensemble models’ performance (eAppendix 5 and eFigure 3 in Supplement 1).

## Results

### Challenge Summary

**Figure 2.**
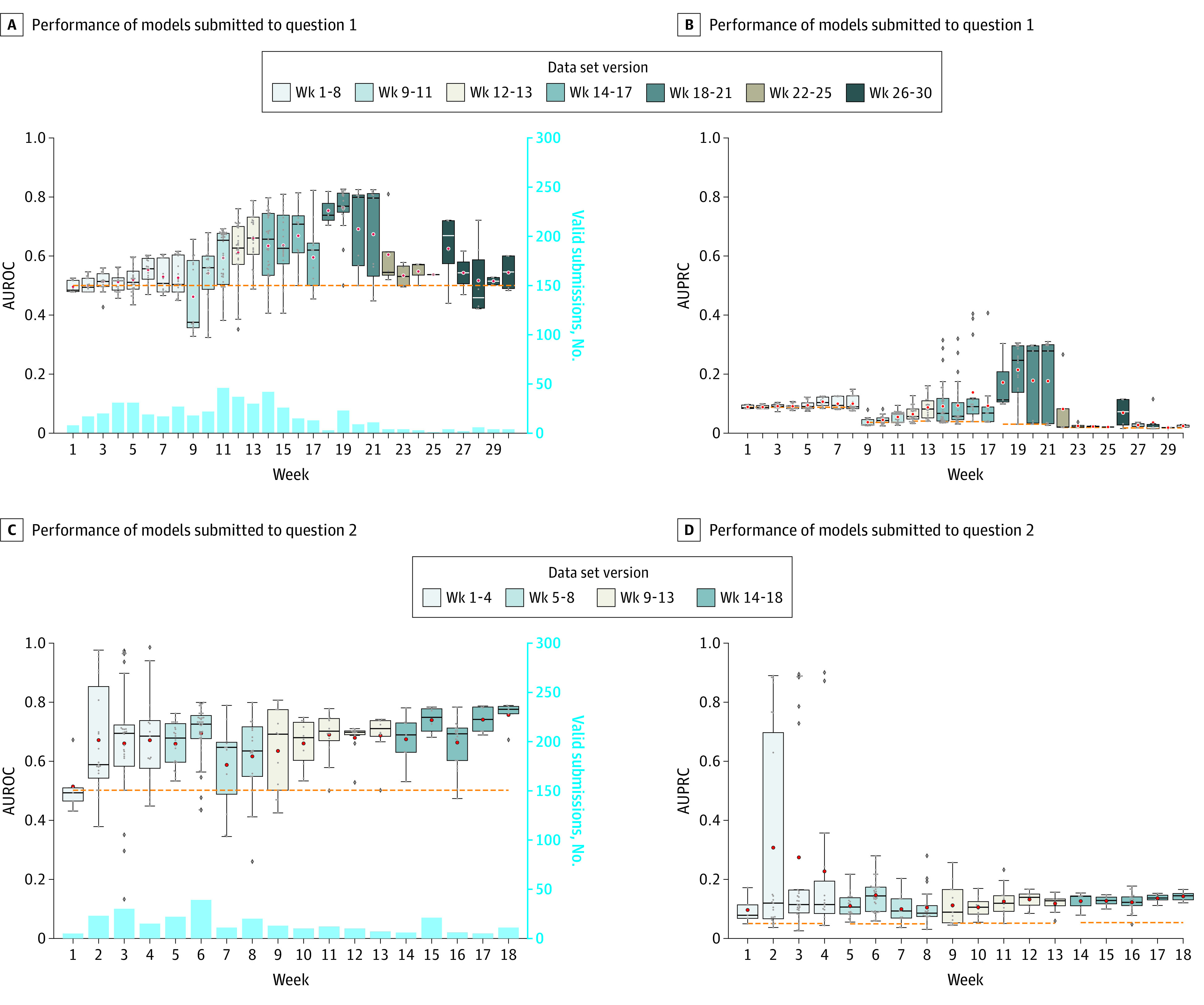
Performance of Models Submitted to Challenge Questions During the Challenge A, Area under the receiver operating characteristic curve (AUROC) of models submitted to question 1 every week. B, Area under the precision recall curve (AUPRC) of models submitted to question 1 every week. C, AUROC of models submitted to question 2 every week. D, AUPRC of models submitted to question 2 every week. Light blue bars in panels A and C show the number of valid submissions to questions 1 and 2 weekly. Data sets were named by the week of the challenge when it is in use. The horizontal dashed line is the performance baseline: for AUROC it is always 0.5 and for AUPRC it is the prevalence of positive patients in each evaluation data set. From bottom to top, the box indicates the 25th to 75th percentile; the error bars indicate the minimum and maximum, respectively, excluding the outliers; the diamonds indicate outliers, the circles indicate mean values; and the horizontal line in each box is the median value.

We hosted a continuously benchmarked community challenge to stimulate the development of machine learning methods for addressing clinical questions around COVID-19. This challenge had 482 registered participants from 90 teams, with 26 teams successfully contributing submissions to at least 1 of the challenge questions. We had 369 valid submissions scored on the Q1 challenge data set and 232 on the Q2 data set. During this challenge, Q1 ran for 30 weeks, with the challenge data set increasing from 9100 patients to 89 600 patients through 6 data updates; Q2 ran for 18 weeks, with the challenge data set increasing from 1700 patients to 2200 patients through 4 data updates. For Q1, the AUROC of the best-performing model was 0.827 and the AUPRC of the best-performing model was 0.303 on the data set version Week 18-21. For Q2, the best AUROC was 0.982 and the best AUPRC was 0.897 for the data set version Week 1-4. However, these scores were observed in the first version of the Q2 challenge data set, which was small, and the top team made multiple submissions in the first 4 weeks, presenting a high risk of overfitting. The best Q2 scores after the first challenge data set version were an AUROC of 0.804 and AUPRC of 0.166 on the data set version Week 9-13 (Figure 2; eFigures 1 and 2 and eTables 6 and 7 in Supplement 1).

### Postchallenge Analysis Results

**Figure 3.**
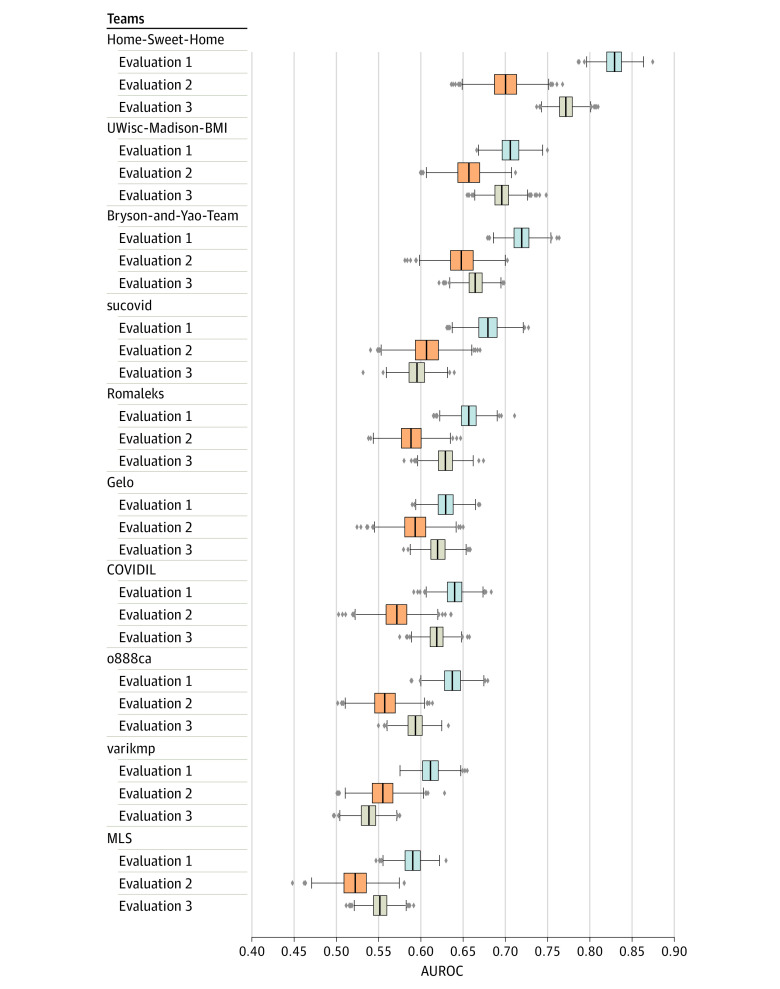
Performance of Question 1 Models on Prospective Data sets Models were trained on the cumulative training data set and evaluated on the temporally split cumulative evaluation data set (evaluation 1, evaluation 2, and evaluation 3) based on the date when patients were tested for COVID-19. The model performance on evaluation 1 was significantly better than the other 2 data sets (*P* < .001). Model performance on evaluation 3 was better than evaluation 2. This might be because evaluation 3 data set’s positive ratio (5.7%) was more similar to the training data set (5.2%) than evaluation 2 (2.5%). From left to right, the box indicates the 25th to 75th percentile; the error bars indicate the minimum and maximum, respectively, excluding the outliers; the diamonds indicate outliers; and the vertical line in each box is the median value. AUROC indicates area under the receiver operating characteristic curve.

The best performance for Q1 on the cumulative data set—defined as data for patients who underwent testing for COVID-19 from March 2, 2020, to January 21, 2021—was an AUROC of 0.776 (95% CI, 0.775-0.777) and an AUPRC of 0.297. We observed considerable variation in models’ AUROCs. We then applied the top 10 retrained models to longitudinally ordered subsets of the cumulative evaluation data set (data sets evaluation 1, evaluation 2, and evaluation 3) to understand how models trained on previous patient data will generalize to future patients. The results for all the top 10 models showed that the performance on the evaluation 1 data set was significantly better than on the evaluation 2 and evaluation 3 data sets (*P* < .001) (Figure 3; eTable 8 in Supplement 1).

**Figure 4.**
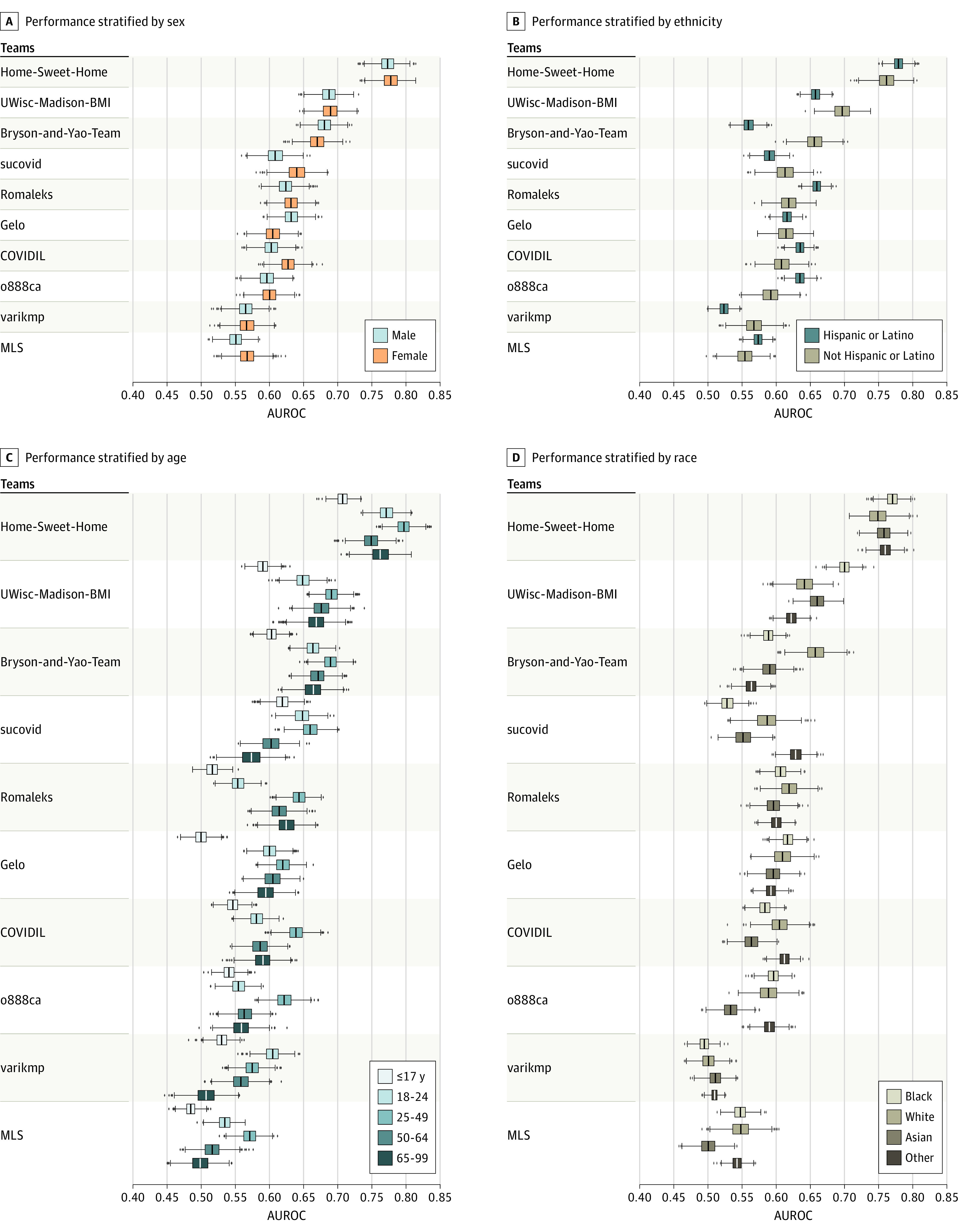
Performance of Question 1 Models in Postchallenge Analysis A, Performance of top 10 question 1 models on subpopulations stratified by sex. B, Performance of top 10 question 1 models on subpopulations stratified by ethnicity. C, Performance of top 10 question 1 models on subpopulations stratified by age. D, Performance of top 10 question 1 models on subpopulations stratified by race. From left to right, the box indicates the 25th to 75th percentile; the error bars indicate the minimum and maximum, respectively, excluding the outliers; the diamonds indicate outliers; and the vertical line in each box is the median value. AUROC indicates area under the receiver operating characteristic curve.

We next explored how the performances of models might vary across different demographic traits. Splitting the cumulative evaluation data set based on sex, 7 of the top 10 teams had significantly better model performance on female subgroups compared with male subgroups (Figure 4A). When splitting by patient age, 8 of the top 10 teams had the lowest prediction performance on the youngest group (≤17 years) and 9 had the highest prediction performance on 25- to 49-year-old patient groups (*P* < .001) (Figure 4C; eTable 8 in Supplement 1). The Pearson correlation coefficient of the top 10 models’ mean AUROC for each age subgroup to the subgroup data set size was 0.849.

The top 10 models did not show a consistent pattern of model performance on the subdata sets split based on ethnicity (Hispanic or Latino and not Hispanic or Latino) or race (Black, White, Asian, and other [the category of “other” was taken from the database, with no further breakdown of specific race and ethnicity categories available]). Among the top 3 teams, the first team (“Home-Sweet-Home”) outperformed the second team (“UWisc-Madison-BMI”) and third team (“Bryson-and-Yao-Team”) in all race groups. However, the third team outperformed the second team in the White race group (Figure 4B and D).

**Figure 5.**
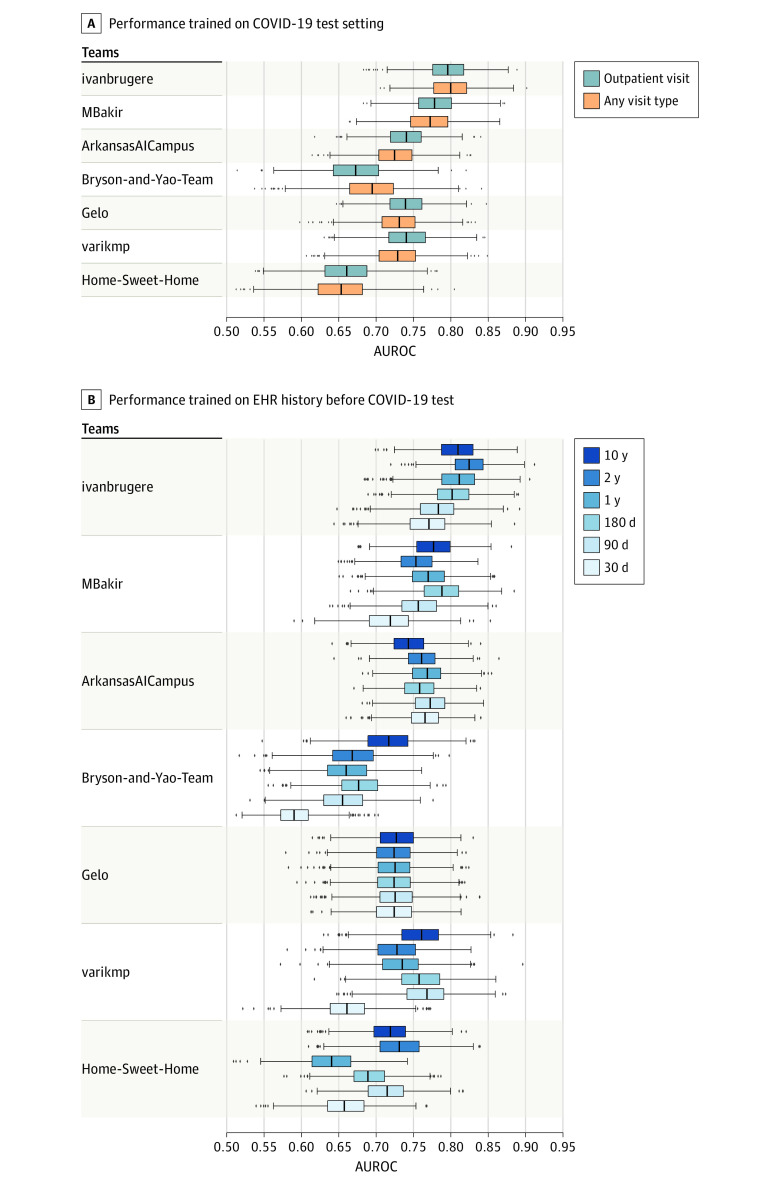
Performance of Question 2 Models in Postchallenge Analysis A, Performance of question 2 models for 21-day hospitalization prediction trained on all patients tested for COVID-19 vs only patients tested for COVID-19 in outpatient settings. B, Performance of question 2 models when trained on different lengths of electronic health record (EHR) history prior to the COVID-19 test. From left to right, the box indicates the 25th to 75th percentile; the error bars indicate the minimum and maximum, respectively, excluding the outliers; the diamonds indicate outliers; and the vertical line in each box is the median value. AUROC indicates area under the receiver operating characteristic curve.

When Q2 models were retrained and evaluated on the cumulative data set, the best AUROC achieved was 0.796 (95% CI, 0.794-0.798), with an AUPRC of 0.188. We asked whether the models could be generalized to patients who received positive COVID-19 test results during all visit types, not just outpatient settings. When the Q2 models were trained and applied to patients who received positive test results in either inpatient or outpatient settings, 4 of 7 models’ performances decreased, and only 1 observed performance increased significantly compared with the prediction for only outpatient patients (eTable 9 in Supplement 1). This finding suggests that hospitalization prediction for patients who underwent testing for COVID-19 during non–outpatient visits, such as patients who were already inpatient for non–COVID-19 health conditions, were more difficult to predict correctly and patient data were noisier and clinically more ambiguous (Figure 5A).

We next tested whether truncating the length of the pre–COVID-19 EHR history made available to prediction models would be associated with model performance. We removed EHR records in 30-day increments up to 10 years before patients’ COVID-19 testing date in both the training and evaluation data sets. We found that model performances did not consistently increase as more EHR clinical history was provided, except for Ivanbrugere’s model, which showed increasing performance as more clinical history became available, up to 2-year data (Figure 5B).

### Top-Performing Methods

We analyzed the top 3 teams’ models for each question to shed light on the features and methods used by participants (eTable 10 in Supplement 1). Model details are included in eAppendix 6 in Supplement 1. The top teams used both a data-driven approach and preselection based on clinical knowledge to select features. Boosting methods were the most popular top-performing algorithms. We asked physicians to review the top features selected by models (eTables 11 and 12 in Supplement 1) to assess if the top features selected by machine learning models were interpretable. Some features appeared to be mechanistically associated with COVID-19, such as loss of smell, cough, fever, and leukocyte count for COVID-19 diagnosis prediction and oxygen saturation, asthma exacerbation diagnosis, acute renal failure, and abnormal coagulation test results for hospitalization prediction. Other features, including serum CO_2_, hemoglobin and hematocrit, albumin, and edema, were selected by the models but did not have a known connection with COVID-19.

### Ensemble Model Performance

We next developed an ensemble model to assess whether combining models could achieve better performance compared with any single model (see Methods). Applying the Q1 ensemble model combining the top 10 models to the ensemble validation data set resulted in higher AUROC performance compared with any single model, with an AUROC of 0.714 (95% CI, 0.713-0.715) and AUPRC of 0.106, compared with Q1 best individual model’s AUROC of 0.699 (95% CI, 0.698-0.700) and AUPRC of 0.112. When stratifying the ensemble validation data set based on demographic profile, the Q1 ensemble model outperformed the best individual model in 10 of total 13 subgroups significantly (eFigure 4 and eTable 13 in Supplement 1). The Q2 ensemble model, which combined the top 7 teams, reached an AUROC of 0.740 (95% CI, 0.739-0.742) and AUPRC of 0.286, compared with Q2 best individual model’s AUROC of 0.772 (95% CI, 0.771-0.774) and AUPRC of 0.193.

## Discussion

In most common research cases, access to patient data is restricted to researchers affiliated with health institutions and the turnaround time to have projects reviewed by institutional review boards can often lead to a delay between the data being available and the study being conducted. These delays and barriers yield missed opportunities for research and impact in time-critical scenarios such as the COVID-19 pandemic. Our citizen science challenge provided a paradigm for sharing up-to-date patient data with those who otherwise would not have access to that data. In this challenge, 482 participants from 7 countries were engaged in training predictive models that could aid clinical decisions and alleviate clinical burden as the COVID-19 pandemic overwhelmed health care institutions. We conducted prospective evaluation and subpopulation analysis of models we received during the postchallenge study. After this study, we will continue operating the platform to support the evaluation of methods on challenge data sets.

We launched 2 questions in this challenge for predicting COVID-19 test results and hospitalization to assess performance of methods, to replicate results from other sites, and to identify key features for prediction. These 2 questions were most suitable for the beginning of the pandemic when test supplies and hospital resources needed to be prioritized. With this continuous benchmarking platform constructed, computational resources provisioned, and hundreds of data scientists engaged, we can point these resources at the next urgent question such as predictions of COVID-19 mortality risk, vaccine effectiveness, and the long-term effects of COVID-19.

We improved this EHR DREAM challenge from a previously fixed data set and time-limited submission quota to data sets that were updated and interrogated over time. The successful operation of the continuous benchmarking challenge demonstrated the flexibility and scalability of the model-to-data approach. This approach proved to have 3 benefits: (1) it protected the patient data while enabling model development on private data; (2) it forced model developers to standardize their models, enabling model transferability and reproducibility for rigorous evaluation; and (3) it enabled an unbiased third party to evaluate these standardized models on previously unseen data.

We saw performance degradation on the temporally evolving data set, indicating limitations in the models’ generalizability on prospective data. However, this performance degradation was expected, given the rapid changes to the challenge data set caused by ever-changing clinical practice and variance in age distribution and prevalence of COVID-19–positive individuals. We observed better model performance in the female group compared with the male group, which could be owing to more female patients than male patients and more EHR history data for the female patients in the cumulative data set. We observed that model performance for the 25- to 49-year-old age group was the best and for the age group 17 years or younger was the worst among all age groups. This finding was consistent with the number of patients in the 2 age groups, in that the 25- to 49-year-old group was the largest and the group 17 years or younger was the smallest. However, with White patients making up most of the data set, the model performance on the White group was not always better than the other race groups, indicating that COVID-19 diagnosis prediction for White patients was difficult even with more training samples for race. We also identified that top teams could have inferior model performance on some subpopulations compared with other teams who ranked lower. This finding could be ameliorated with a model ensemble based on the strength of each team to maximize prediction accuracy. The Q1 ensemble model outperformed the best individual model in most demographic subgroups.

The high-performing models we received in the challenge indicate potential clinical utility. To achieve that, we will need to further test the generalizability of those models in a larger and multisite data set (eg, National COVID Cohort Collaborative data^18^) and incorporate the models into a live clinical workflow setting for providing clinical decision support.

### Limitations

This study has some limitations. The continuous benchmarking challenge in itself has led to the identification of several limitations. Data quality was difficult to maintain with regular updates. Data duplicates existed in some versions of the challenge data set. In addition, compared with conventional challenges that have a fixed time frame, models were more at risk of overfitting to the data as the number of allowed submissions increased over time. We also noticed that challenge models may be biased against 1 or more subpopulations, and it is not always the case that this is caused by the training data size; it could be caused by cultural and behavioral differences and requires further investigation.

## Conclusions

We succeeded in operating a continuous benchmarking challenge to share up-to-date COVID-19 EHR patient data with a worldwide data science community. The benchmarking challenge provided an unbiased evaluation of models submitted by participants. Top models achieved high accuracy in predicting COVID-19 diagnosis results and hospitalization, indicating potential for clinical implementation. Across submitted models, we observed discrepancies of performance in this temporally evolving data set and among demographic subpopulations (sex, age, race, and ethnicity), indicating the existence of potential bias in machine learning approaches, which warrants attention prior to implementation of such models in clinical practice.

## Notes

Supplement 1. **eAppendix 1.** Challenge Timeline **eAppendix 2.** Challenge Dataset **eTable 1.** Demographics Decomposition for Question 1 Challenge Datasets **eTable 2.** Demographics Decomposition for Question 2 Challenge Datasets **eTable 3.** Demographics Decomposition for the Cumulative Dataset, Temporal-Split Cumulative Evaluation Dataset (Evaluation 1, Evaluation 2 and Evaluation 3) and Ensemble Validation Dataset **eAppendix 3.** Computational Resources for the Challenge **eAppendix 4.** Model Selection Criteria **eTable 4.** The Performance Ranking of Models Submitted to Question 1 **eTable 5.** The Performance Ranking of Models Submitted to Question 2 **eFigure 1.** Weekly Best-Performing Models Submitted to Question 1 **eTable 6.** Best Model Performance Reached Weekly for Question 1 **eFigure 2.** Weekly Best-Performing Models Submitted to Question 2 **eTable 7.** Best Model Performance Reached Weekly for Question 2 **eTable 8.** Subpopulation Post-Challenge for Question 1 **eTable 9.** Subpopulation Post-Challenge for Question 2 **eTable 10.** Analysis for Top Models Submitted to Challenge Question 1 and Question 2 **eTable 11.** Top 10 Features for Question 1 **eTable 12.** Top 10 Features for Question 2 **eAppendix 5.** Ensemble Model **eFigure 3.** Ensemble Model Diagram **eFigure 4.** Model Performance Comparison Between Question 1 Ensemble Model and Question 1 Best Individual Model on Demographics Subgroups Using Ensemble Validation Dataset **eTable 13.** Model Performance (AUROCs and 95% CI of AUROCs) Comparison Between Question 1 Ensemble Model and Question 1 Best Individual Model on Demographics Subgroups **eAppendix 6.** Top Teams’ Model Description

Supplement 2. **Nonauthor Collarborators.** DREAM Challenge Consortium
