# Supplementary material for: A Continuously Benchmarked and Crowdsourced Challenge for Rapid Development and Evaluation of Models to Predict COVID-19 Diagnosis and Hospitalization
Source: JAMA Netw Open. 2021 Oct 11;4(10):e2124946. doi: 10.1001/jamanetworkopen.2021.24946 (PMC8506231; doi:10.1001/jamanetworkopen.2021.24946)
Supplement: Supplement 1. — eAppendix 1. Challenge Timeline eAppendix 2. Challenge Dataset eTable 1. Demographics Decomposition for Question 1 Challenge Datasets eTable 2. Demographics Decomposition for Question 2 Challenge Datasets eTable 3. Demographics Decomposition for the Cumulative Dataset, Temporal-Split Cumulative Evaluation Dataset (Evaluation 1, Evaluation 2 and Evaluation 3) and Ensemble Validation Dataset eAppendix 3. Computational Resources for the Challenge eAppendix 4. Model Selection Criteria eTable 4. The Performance Ranking of Models Submitted to Question 1 eTable 5. The Performance Ranking of Models Submitted to Question 2 eFigure 1. Weekly Best-Performing Models Submitted to Question 1 eTable 6. Best Model Performance Reached Weekly for Question 1 eFigure 2. Weekly Best-Performing Models Submitted to Question 2 eTable 7. Best Model Performance Reached Weekly for Question 2 eTable 8. Subpopulation Post-Challenge for Question 1 eTable 9. Subpopulation Post-Challenge for Question 2 eTable 10. Analysis for Top Models Submitted to Challenge Question 1 and Question 2 eTable 11. Top 10 Features for Question 1 eTable 12. Top 10 Features for Question 2 eAppendix 5. Ensemble Model eFigure 3. Ensemble Model Diagram eFigure 4. Model Performance Comparison Between Question 1 Ensemble Model and Question 1 Best Individual Model on Demographics Subgroups Using Ensemble Validation Dataset eTable 13. Model Performance (AUROCs and 95% CI of AUROCs) Comparison Between Question 1 Ensemble Model and Question 1 Best Individual Model on Demographics Subgroups eAppendix 6. Top Teams’ Model Description [file jamanetwopen-e2124946-s001.pdf]

Supplementary Online Content

Yan Y, Schaffter T, Bergquist T, et al; DREAM Challenge Consortium. A continuously benchmarked and crowdsourced challenge for rapid development and evaluation of models to predict COVID-19 diagnosis and hospitalization. *JAMA Netw Open.* 2021;4(10):e2124946. doi:10.1001/jamanetworkopen.2021.24946

eAppendix 1. Challenge Timeline
eAppendix 2. Challenge Dataset
eTable 1. Demographics Decomposition for Question 1 Challenge Datasets
eTable 2. Demographics Decomposition for Question 2 Challenge Datasets
eTable 3. Demographics Decomposition for the Cumulative Dataset, Temporal-Split Cumulative Evaluation Dataset (Evaluation 1, Evaluation 2 and Evaluation 3) and Ensemble Validation Dataset
eAppendix 3. Computational Resources for the Challenge
eAppendix 4. Model Selection Criteria
eTable 4. The Performance Ranking of Models Submitted to Question 1
eTable 5. The Performance Ranking of Models Submitted to Question 2
eFigure 1. Weekly Best-Performing Models Submitted to Question 1
eTable 6. Best Model Performance Reached Weekly for Question 1
eFigure 2. Weekly Best-Performing Models Submitted to Question 2
eTable 7. Best Model Performance Reached Weekly for Question 2
eTable 8. Subpopulation Post-Challenge for Question 1
eTable 9. Subpopulation Post-cHallenge for Question 2
eTable 10. Analysis for Top Models Submitted to Challenge Question 1 and Question 2
eTable 11. Top 10 Features for Question 1
eTable 12. Top 10 Features for Question 2
eAppendix 5. Ensemble Model
eFigure 3. Ensemble Model Diagram
eFigure 4. Model Performance Comparison Between Question 1 Ensemble Model and Question 1 Best Individual Model on Demographics Subgroups Using Ensemble Validation Dataset
eTable 13. Model Performance (AUROCs and 95% CI of AUROCs) Comparison Between Question 1 Ensemble Model and Question 1 Best Individual Model on Demographics Subgroups
eAppendix 6. Top Teams' Model Description

This supplementary material has been provided by the authors to give readers additional information about their work.

© 2021 Yan Y et al. *JAMA Network Open.*

eAppendix 1. Challenge Timeline

We launched the COVID-19 EHR DREAM challenge Question 1 (Q1) on May 6 2020 and Question 2 (Q2) on August 19 2020. When we started the challenge on May 6 2020, there were insufficient patients to split the data into training and evaluation datasets, so we initially started an open phase for Q1 where we only provided an evaluation dataset. We encouraged participants to submit models using either rule-based models or pre-trained models using data from other sources like COVID-19 Electronic Health Record (EHR) data from their own institutes' data warehouse or publicly available data. This open phase (dataset version "Week 1-8") for Q1 lasted 8 weeks until we enabled model training on July 6 2020. The data for Q2 had enough patients to start the challenge question with a training dataset provided. Q1 was run for 30 weeks and Q2 was run for 18 weeks until December 23 2020.

© 2021 Yan Y et al. *JAMA Network Open.*

eAppendix 2. Challenge Dataset

COVID-19 Dataset

The challenge data was derived from the University of Washington (UW) Enterprise Data Warehouse (EDW) and contained EHR data from patients tested for COVID-19 at multiple medical sites across the UW Medicine Health system including UW Medical Center, Northwest Hospital, Harborview Medical Center, Neighborhood Clinics, and Seattle Cancer Care Alliance. The data contained visit history, laboratory results, demographic data, diagnosis codes, and procedures performed. UW Medicine Research IT converted the EHR data from the EDW to a standardized Observational Medical Outcomes Partnerships Common Data Model (OMOP CDM v5.3.1). Historical health data for patients who contracted COVID-19 during the pandemic ranged as far back as 2010.

Challenge dataset

We ran the COVID-19 EHR DREAM challenge as a continuous benchmarking exercise where the datasets were updated every 2-5 weeks to incorporate new patients and update existing patients' clinical trajectory. We curated two sub-datasets (diagnostic Q1 challenge dataset and prognostic Q2 challenge dataset) separately for the two challenge questions for the purpose of model training and evaluation. The Q1 challenge dataset involving all patients who received a COVID-19 test by the date when each data update was conducted, was split into training and evaluation datasets using a temporal ordering where the EHR data for the most recent 20% of patients tested for COVID-19 were included in the evaluation dataset and the remaining 80% of patients were included in the training dataset. The Q2 challenge dataset included only patients who tested positive for COVID-19 at an outpatient setting by the date when data update was conducted. This data was randomly split into training (70%) and evaluation datasets (30%) in order to maintain the same true positive prevalence (hospitalization within 21 days versus all patients tested positive during an outpatient visit). Gold standards for the training data were provided for model training and gold standards for evaluation dataset were hidden by the challenge organizers for scoring. For both the Q1 challenge dataset and Q2 challenge dataset, EHR data after each patient's COVID-19 test were removed. We incorporated new patients (i.e., patients who were not included in previous data update) into the evaluation datasets and made sure no patient data existing in the training dataset of previous versions was included in later evaluation dataset versions. Q1 challenge dataset has 6 versions over 30 weeks and Q2 challenge dataset has 4 versions over 18 weeks. See eTable 1 and eTable 2 for details about the challenge datasets.

Synthetic data

We also curated a synthetic dataset which was adapted from the SynPuf (Synthetic OMOP dataset) to accurately reflect the distribution and size of the UW COVID-19 patient dataset. We randomly sampled clinical terms and concepts that appeared in more than 100 person's clinical records and populated the synthetic data with these terms. To better capture the record distribution, we created synthetic visit records and added them to individual patients until the synthetic record distribution resembled the real data record distribution, making sure that the number of patients with one visit, 10 visits, 100 visits was similar between the two datasets.

© 2021 Yan Y et al. *JAMA Network Open.*

eTable 1. Demographics Decomposition for Question 1 Challenge Datasets

Dataset version	Week 1-8	Week 9-11		Week 12-13		Week 14-17		Week 18-21		Week 22-25		Week 26-30	
Demographic	Eval	Train	Eval	Train	Eval	Train	Eval	Train	Eval	Train	Eval	Train	Eval
Age (%)													
0-17	1.64	2.09	3.95	2.50	5.43	2.86	5.79	3.26	4.86	3.50	4.41	3.68	3.71
18-24	5.85	6.79	17.88	9.05	9.21	9.10	9.44	9.14	9.24	9.14	12.47	9.26	10.97
25-49	41.41	40.71	36.81	39.88	40.45	39.93	39.25	39.96	35.91	39.65	35.82	39.45	35.08
50-64	27.27	26.33	22.10	25.48	23.69	25.28	23.64	25.05	25.89	25.04	24.34	24.97	25.17
65-99	23.83	24.08	19.26	23.08	21.22	22.82	21.88	22.58	24.09	22.67	22.96	22.64	25.07
Gender (%)													
Female	53.07	51.80	51.17	51.57	50.89	51.56	50.44	51.40	50.74	51.35	51.02	51.28	51.57
Male	46.90	48.13	48.71	48.36	49.07	48.38	49.52	48.54	49.24	48.60	48.97	48.67	48.41
Other/Nan	0.03	0.07	0.12	0.07	0.04	0.06	0.04	0.06	0.02	0.05	0.01	0.05	0.02
Race (%)													
White	66.54	65.75	62.74	65.23	63.78	65.20	62.68	64.93	63.45	64.75	65.37	64.71	67.71
Asian	9.25	8.88	9.60	9.07	8.14	8.99	8.46	8.91	9.83	9.02	10.80	9.18	13.77
Black	9.70	10.01	8.55	9.73	9.02	9.70	8.67	9.56	10.22	9.57	8.50	9.56	10.46
Other/Nan	14.51	15.36	19.11	15.97	19.06	16.10	20.18	16.61	16.50	16.66	15.32	16.55	8.06
Covid-19 test (%)													
Positive	8.77	4.78	3.66	4.67	4.10	4.67	3.92	4.61	3.06	4.60	1.94	4.50	1.80
Negative	91.23	95.22	96.34	95.33	95.90	95.33	96.08	95.39	96.94	95.40	98.06	95.50	98.20
population (count)	9134	35276	8464	45106	11186	49891	12325	58175	14054	64836	16008	71792	17841
Earliest COVID measurement date (2020)			06-20		07-09		07-19		08-06		08-23		09-12

© 2021 Yan Y et al. *JAMA Network Open.*

eTable 2. Demographics Decomposition for Question 2 Challenge Datasets

Dataset version	Week 1-4		Week 5-8		Week 9-13		Week 14-18	
Demographic	Train	Eval	Train	Eval	Train	Eval	Train	Eval
Age (%)								
0-17	5.84	4.66	6.13	6.53	6.02	6.66	5.96	6.56
18-24	17.91	17.54	16.91	17.99	17.62	18.15	17.45	18.18
25-49	43.80	44.40	45.01	42.83	44.20	42.36	44.35	42.47
50-64	20.54	23.51	20.82	23.41	20.82	23.45	20.69	23.40
65-99	11.91	9.89	11.12	9.24	11.34	9.38	11.55	9.39
Gender (%)								
Female	50.68	51.87	50.27	51.91	50.10	51.13	49.87	51.12
Male	49.24	48.13	49.66	48.09	49.84	48.87	50.06	48.88
Other/Nan	0.08	0.00	0.07	0.00	0.06	0.00	0.06	0.00
Race (%)								
White	48.16	43.21	46.47	41.88	46.97	41.60	46.83	41.64
Asian	6.69	9.94	6.73	9.74	6.85	10.17	33.66	10.03
Black	11.51	15.49	12.08	15.10	12.39	15.25	12.53	15.50
Other/Nan	33.63	31.36	34.73	33.28	33.79	32.97	6.98	32.83
21-day hospitalization (%)								
Positive	5.04	5.04	4.99	4.94	5.12	5.14	5.52	5.37
Negative	94.96	94.96	95.01	95.06	94.88	94.86	94.48	94.63
population (count)	1251	536	1484	628	1561	661	1576	671

© 2021 Yan Y et al. *JAMA Network Open.*

eTable 3. Demographics Decomposition for the Cumulative Dataset, Temporal-Split Cumulative Evaluation Dataset (Evaluation 1, Evaluation 2 and Evaluation 3) and Ensemble Validation Dataset

	Demographic	cumulative training dataset	cumulative evaluation dataset	Evaluation 1	Evaluation 2	Evaluation 3	ensemble validation dataset
	Age (%)						
	0-17	3.05	4.51	5.60	3.75	4.18	5.58
	18-24	9.06	9.91	9.82	11.50	8.40	6.93
	25-49	39.89	37.73	38.03	34.92	40.25	38.23
	50-64	25.21	24.34	24.27	25.00	23.76	23.9
	65-99	22.79	23.51	22.28	24.83	23.40	25.36
	Gender (%)						
	Female	51.47	51.07	50.76	51.68	50.77	52.06
	Male	48.47	48.90	49.21	48.31	49.17	47.9
	Other/Nan	0.06	0.03	0.03	0.01	0.06	0.04
	Race (%)						
	White	64.66	65.14	61.91	67.79	65.72	68.00
	Asian	8.89	10.30	9.85	10.48	10.58	11.17
	Black	9.51	8.64	9.15	8.04	8.73	8.14
	Other/Nan	16.93	15.92	19.10	13.69	14.97	12.69
	Covid-19 test (%)						
	Positive	5.16	4.02	3.93	2.45	5.68	2.16
	Negative	94.84	95.98	96.07	97.55	94.32	97.84
Q1	Number of patients tested for COVID	54600	53936	17932	18020	17984	12870
	Patients hospitalized within 21 days (%)						
	Positive	5.73	5.10				7.66
	Negative	94.27	94.90				92.34
Q2	Number of patients tested positive for COVID in outpatient setting(count)	1554	1552	---	---	---	208

© 2021 Yan Y et al. *JAMA Network Open.*

eAppendix 3. Computational Resources for the Challenge

For models submitted by participants, we used a Common Workflow Language (CWL) pipeline to coordinate submission queues and automatically download and run Docker images. We provided two computation environments for participants (1) a cloud environment, with access to 32 CPU cores and 249 GB RAM, and (2) a UW on-premises server, with access to 32 CPU cores and 70 GB RAM. Participants were allowed up to 2 hours runtime at each environment.

eAppendix 4. Model Selection Criteria

We received valid submissions (models scoring area under the receiver operating characteristic (AUROC) > 0.5) to Q1 from 18 teams. Over the course of the challenge, teams submitted multiple models that were scored against different versions of the COVID-19 patient datasets. For this analysis, we selected each team's best performing model (highest AUROC), regardless of the data version on which the model achieved its highest score. We re-trained each of the models on the cumulative training dataset and evaluated them on the cumulative evaluation datasets to select the top 10 models used for the analysis. Q1 model ranking is listed in eTable 4. We received valid submissions (models scoring AUROC > 0.5) to Q2 from 7 teams. Q2 model ranking is listed in eTable 5.

AUROC was used as the primary scoring metric for assessing model performance. The Bayes Factor, K, (bootstrapped distributions $n = 1000$) was computed to determine if the AUROCs between two models were consistently different. If two models were found to have a small Bayes Factor ($K < 19$), we used the area under the precision-recall curve (AUPRC) as a tie-breaking metric. Bayes factor was calculated using the number of times the current group won divided by the number of times the comparison group won. E.g., During the bootstrapping for Home-Sweet-Home and UWisc-Madison-BMI, Home-Sweet-Home won 1000 times, UWisc-Madison-BMI won 0 times. So, in eTable 4, the Bayes score of Home-Sweet-Home compared to UWisc-Madison-BMI is Inf.

© 2021 Yan Y et al. *JAMA Network Open.*

eTable 4. The Performance Ranking of Models Submitted to Question 1. Bootstrapping (*n* = 1000, sample size 10000, sampled with replacement) to get a distribution of model performance.

Team	Model Performance on cumulative dataset			
	AUROC	AUROC 95% CI	AUPRC	Bayes Factor*
Home-Sweet-Home	0.7757	(0.7748, 0.7765)	0.2974	Inf
UWisc-Madison-BMI	0.6893	(0.6884, 0.6902)	0.1692	3.07
Bryson-and-Yao-Team	0.6782	(0.6774, 0.6791)	0.0805	499.00
sucovid	0.6250	(0.6240,0.6260)	0.0838	0.75
Romaleks	0.6290	(0.6281, 0.6299)	0.0682	3.65
Gelo	0.6162	(0.6153, 0.6170)	0.0589	1.06
COVIDIL	0.6147	(0.6138, 0.6156)	0.0564	6.52
o888ca	0.5998	(0.5989, 0.6006)	0.0540	19.83
varikmp	0.5661	(0.5652, 0.5670)	0.0504	1.61
MLS	0.5585	(0.5576, 0.5593)	0.0444	

eTable 5. The Performance Ranking of Models Submitted to Question 2. Bootstrapping (*n* = 1000, sample size 1000, sampled with replacement) to get a distribution of model performance.

Team	Model Performance on cumulative dataset			
	AUROC	AUROC 95% CI	AUPR	Bayes Factor
ivanbrugere	0.7963	(0.7943,0.7982)	0.1875	3.20
MBakir	0.7759	(0.7738,0.7780)	0.1783	6.93
ArkansasAICampus20	0.7407	(0.7388, 0.7426)	0.1617	12.17
Bryson-and-Yao-Team	0.6701	(0.6674, 0.6728)	0.1593	0.11
Gelo	0.7395	(0.7376, 0.7415)	0.1403	0.93
varikmp	0.7411	(0.7389, 0.7432)	0.1308	15.95
Home-Sweet-Home	0.6588	(0.6563, 0.6613)	0.0944	-

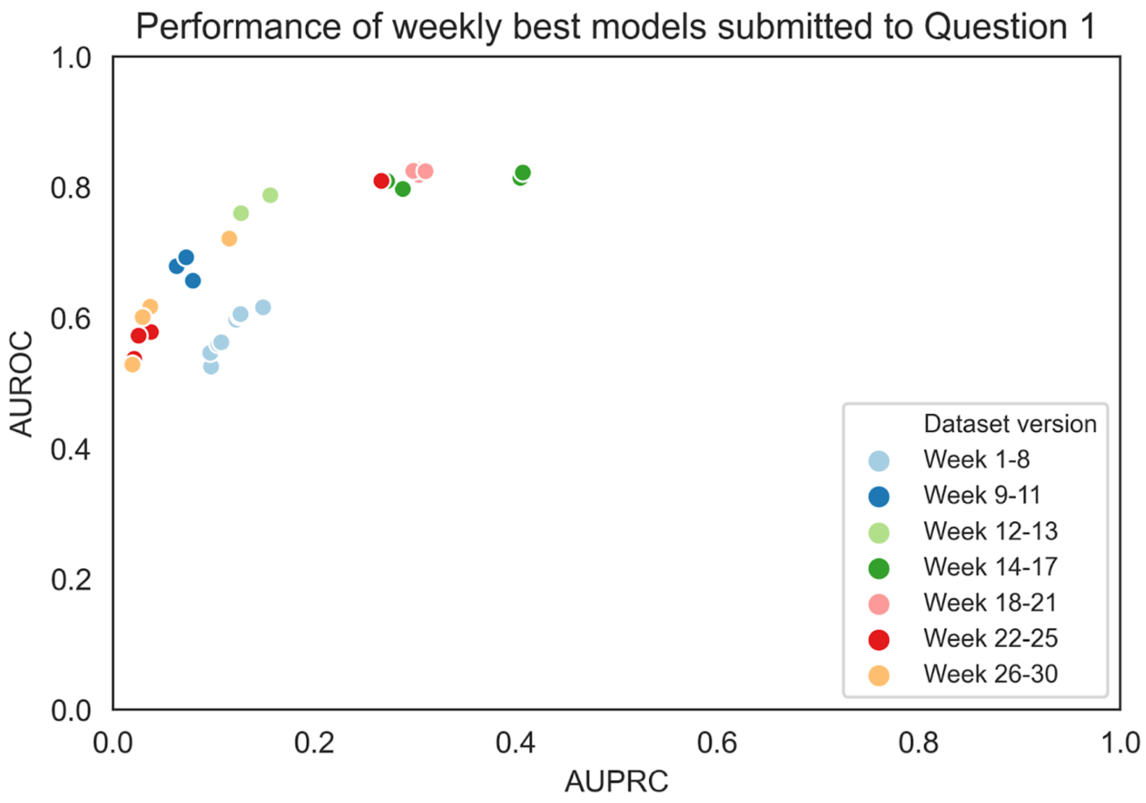

eFigure 1. Weekly Best-Performing Models Submitted to Question 1

© 2021 Yan Y et al. *JAMA Network Open.*

eTable 6. Best Model Performance Reached Weekly for Question 1

Week	AUROC	AUPRC	Dataset, version
1	0.525	0.097	Week 1-8
2	0.546	0.097	Week 1-8
3	0.560	0.105	Week 1-8
4	0.562	0.107	Week 1-8
5	0.597	0.122	Week 1-8
6	0.603	0.125	Week 1-8
7	0.606	0.127	Week 1-8
8	0.616	0.149	Week 1-8
9	0.657	0.079	Week 9-11
10	0.679	0.063	Week 9-11
11	0.693	0.073	Week 9-11
12	0.760	0.127	Week 12-13
13	0.788	0.156	Week 12-13
14	0.797	0.288	Week 14-17
15	0.809	0.272	Week 14-17
16	0.814	0.404	Week 14-17
17	0.822	0.407	Week 14-17
18	0.819	0.304	Week 18-21
19	0.827	0.303	Week 18-21
20	0.825	0.298	Week 18-21
21	0.824	0.310	Week 18-21
22	0.810	0.266	Week 22-25
23	0.578	0.038	Week 22-25
24	0.572	0.025	Week 22-25
25	0.537	0.021	Week 22-25
26	0.721	0.115	Week 26-30
27	0.617	0.037	Week 26-30
28	0.721	0.115	Week 26-30
29	0.528	0.019	Week 26-30
30	0.601	0.029	Week 26-30

© 2021 Yan Y et al. *JAMA Network Open.*

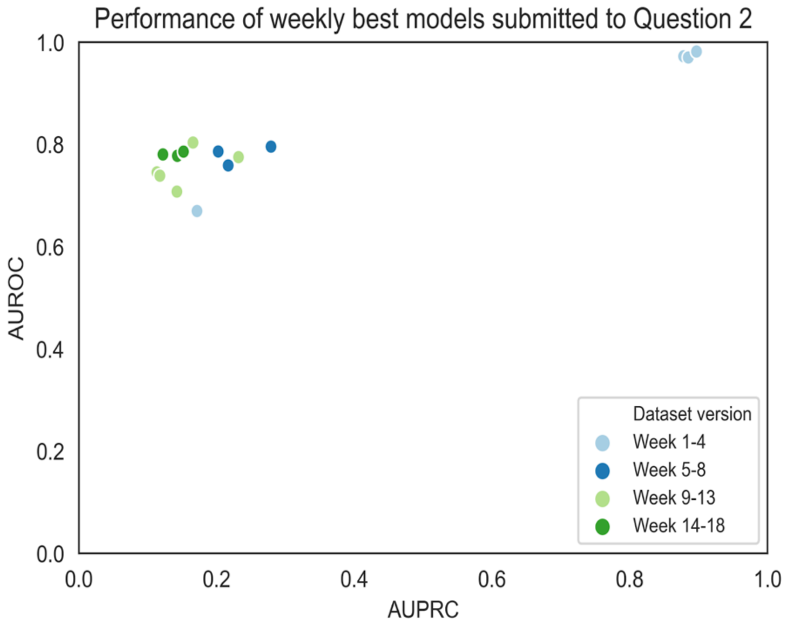

eFigure 2. Weekly Best-Performing Models Submitted to Question 2

© 2021 Yan Y et al. *JAMA Network Open.*

eTable 7. Best Model Performance Reached Weekly for Question 2

Week	AUROC	AUPRC	Dataset version
1	0.670	0.171	Week 1-4
2	0.972	0.878	Week 1-4
3	0.970	0.885	Week 1-4
4	0.982	0.897	Week 1-4
5	0.759	0.217	Week 5-8
6	0.796	0.279	Week 5-8
7	0.786	0.202	Week 5-8
8	0.796	0.279	Week 5-8
9	0.804	0.166	Week 9-13
10	0.745	0.113	Week 9-13
11	0.775	0.232	Week 9-13
12	0.708	0.142	Week 9-13
13	0.739	0.118	Week 9-13
14	0.778	0.143	Week 14-18
15	0.780	0.122	Week 14-18
16	0.780	0.122	Week 14-18
17	0.784	0.152	Week 14-18
18	0.786	0.152	Week 14-18

© 2021 Yan Y et al. *JAMA Network Open.*

eTable 8. Subpopulation Post-Challenge for Question 1. AUROCs and 95% CI of AUROCs on sub-evaluation groups for Question 1.

Team	Prospective evaluation			Gender		Age				
	Evaluation 1	Evaluation 2	Evaluation 3	Female	Male	0-17	18-24	25-49	50-64	65-99
Home-Sweet-Home	0.829 (0.828, 0.830)	0.700 (0.699, 0.702)	0.772 (0.771, 0.772)	0.778 (0.777, 0.778)	0.773 (0.772, 0.774)	0.708 (0.707, 0.708)	0.772 (0.771, 0.773)	0.797 (0.796, 0.798)	0.749 (0.748, 0.750)	0.763 (0.762, 0.764)
UWisc-Madison-BMI	0.706 (0.705, 0.707)	0.656 (0.655, 0.657)	0.696 (0.695, 0.696)	0.689 (0.688, 0.690)	0.687 (0.686, 0.688)	0.590 (0.590, 0.591)	0.649 (0.649, 0.650)	0.692 (0.691, 0.692)	0.677 (0.676, 0.678)	0.669 (0.668, 0.670)
Bryson-and-Yao-Team	0.719 (0.719, 0.720)	0.648 (0.647, 0.649)	0.665 (0.664, 0.665)	0.670 (0.669, 0.671)	0.681 (0.680, 0.681)	0.603 (0.603, 0.604)	0.664 (0.663, 0.665)	0.690 (0.689, 0.691)	0.671 (0.670, 0.671)	0.664 (0.663, 0.665)
sucovid	0.679 (0.678, 0.680)	0.607 (0.606, 0.608)	0.595 (0.595, 0.596)	0.640 (0.639, 0.641)	0.609 (0.608, 0.610)	0.619 (0.618, 0.620)	0.648 (0.648, 0.649)	0.660 (0.659, 0.661)	0.602 (0.601, 0.603)	0.574 (0.573, 0.575)
Romaleks	0.657 (0.656, 0.657)	0.588 (0.587, 0.589)	0.629 (0.628, 0.630)	0.632 (0.631, 0.633)	0.624 (0.623, 0.625)	0.516 (0.516, 0.517)	0.554 (0.553, 0.555)	0.644 (0.643, 0.644)	0.615 (0.614, 0.615)	0.625 (0.624, 0.626)
Gelo	0.629 (0.628, 0.630)	0.593 (0.592, 0.594)	0.620 (0.619, 0.621)	0.605 (0.604, 0.606)	0.632 (0.631, 0.633)	0.500 (0.499, 0.501)	0.600 (0.600, 0.601)	0.620 (0.619, 0.621)	0.605 (0.604, 0.606)	0.595 (0.594, 0.596)
COVIDIL	0.640 (0.639, 0.641)	0.571 (0.570, 0.572)	0.618 (0.618, 0.619)	0.627 (0.626, 0.628)	0.603 (0.602, 0.603)	0.547 (0.546, 0.547)	0.582 (0.581, 0.582)	0.640 (0.639, 0.640)	0.587 (0.586, 0.588)	0.590 (0.589, 0.591)
o888ca	0.638 (0.637, 0.638)	0.557 (0.556, 0.558)	0.593 (0.592, 0.594)	0.600 (0.599, 0.601)	0.596 (0.595, 0.597)	0.541 (0.541, 0.542)	0.555 (0.554, 0.556)	0.622 (0.621, 0.623)	0.564 (0.563, 0.565)	0.559 (0.558, 0.560)
varikmp	0.611 (0.611, 0.612)	0.555 (0.554, 0.556)	0.538 (0.537, 0.539)	0.567 (0.566, 0.568)	0.565 (0.564, 0.566)	0.530 (0.530, 0.531)	0.605 (0.604, 0.606)	0.575 (0.574, 0.576)	0.558 (0.557, 0.559)	0.508 (0.506, 0.509)
MLS	0.590 (0.589, 0.591)	0.522 (0.521, 0.523)	0.552 (0.551, 0.553)	0.567 (0.566, 0.568)	0.550 (0.550, 0.551)	0.485 (0.484, 0.486)	0.535 (0.534, 0.536)	0.572 (0.571, 0.572)	0.516 (0.516, 0.517)	0.499 (0.498, 0.500)

© 2021 Yan Y et al. *JAMA Network Open.*

eTable 9. Subpopulation Post-challenge for Question 2. AUROCs and 95% CI of AUROCs on sub-evaluation groups for Question 2.

Team	Status	
	patients tested positive during an outpatient visit	patients tested positive at any visit types'
ivanbrugere	0.794 (0.792, 0.796)	0.799 (0.797, 0.801)
MBakir	0.776 (0.774, 0.778)	0.773 (0.770, 0.775)
ArkansasAICampus20	0.740 (0.738, 0.741)	0.725 (0.723, 0.727)
Bryson-and-Yao-Team	0.672 (0.669, 0.674)	0.695 (0.692, 0.698)
Gelo	0.741 (0.739, 0.743)	0.728 (0.726, 0.730)
varikmp	0.740 (0.738, 0.742)	0.728 (0.726, 0.731)
Home-Sweet-Home	0.659 (0.656, 0.662)	0.652 (0.649, 0.655)

© 2021 Yan Y et al. *JAMA Network Open.*

eTable 10. Analysis for Top Models Submitted to Challenge Question 1 and Question 2

**Q1 top models**	**Home-Sweet-Home**	**UWisc-Madison-BMI**	**Bryson-and-Yao-Team**
Number of features used	168	55	22
Feature selection	Data driven	pre-selected engineered features	pre-selected engineered features
machine learning model type	LightGBM	LightGBM	Logistic Regression
EHR data cut-off date	After January 1st, 2020 (for some tables)	Only after February 1st, 2020	No cut-off date
OMOP table used for building the model	measurement, condition occurrence, observation, drug exposure, device exposure, procedure occurrence, visit occurrence, person, goldstandard (for training)	person, condition occurrence, measurement	person, condition occurrence, measurement
**Q2 top models**	**Ivanbrugere**	**ArkansasAICampus**	**Bryson-and-Yao-Team**
Number of features used	6209	15232	49
Feature selection	data-driven	data-driven + pre-selected engineered features	data-driven + pre-selected engineered features
Machine learning model type	Boosted trees (catboost)	Ensemble of random trees (ExtraTreesClassifier)	Logistic Regression
EHR data cut-off date	No: all dates	No: all dates	Condition: After January 1st 2015 Measurement: After January 1st 2019
OMOP table used for building the model	condition occurrence, observation, measurement, drug exposure, person, visit occurrence	condition, drug exposure, measurement, observation, person	person, condition occurrence, measurement

© 2021 Yan Y et al. *JAMA Network Open.*

eTable 11. Top 10 Features for Question 1

	Home-Sweet-Home	UWisc-Madison-BMI	Bryson-and-Yao-Team
1	Age	Ethnicity	Female
2	Count of Telephone call to a patient after 2020/01/01, if not existing, on or before 2020/01/01	Most recent value of Leukocytes [#/volume] in Blood by Automated count after 2020-02-01	Loss of sense of smell
3	Count of outpatient visit after 2020/01/01, if not existing, on or before 2020/01/01	Age	Hispanic
4	Mean measurement of Mean Albumin [Mass/volume] in Serum or Plasma after 2020/01/01, if not existing, on or before 2020/01/01	Most recent value of Creatinine [Mass/volume] in Serum or Plasma after 2020-02-01	Not Hispanic or Latino
5	Mean measurement of diastolic blood pressure after 2020/01/01, if not existing, on or before 2020/01/01	Presence of cough after 2020-02-01	Cough
6	Mean measurement of carbon dioxide, total [Moles/volume] in Serum or Plasma after 2020/01/01, if not existing, on or before 2020/01/01	Presence of fever after 2020-02-01	Race-unknown
7	Count of viral pneumonia after 2020/01/01, if not existing, on or before 2020/01/01	Most recent value of hematocrit after 2020-02-01	Fever
8	Maximum measurement of Systolic blood pressure after 2020/01/01, if not existing, on or before 2020/01/01	Presence of Viral pneumonia after 2020-02-01	Pneumonia
9	Minimum measurement of Systolic blood pressure after 2020/01/01, if not existing, on or before 2020/01/01	Most recent value of Hemoglobin [Mass/volume] in Blood after 2020-02-01	Diastolic blood pressure
10	Mean measurement of Systolic blood pressure after 2020/01/01, if not existing, on or before 2020/01/01	Sex	White-race

© 2021 Yan Y et al. *JAMA Network Open.*

eTable 12. Top 10 Features for Question 2

	**Ivanbrugere**	**ArkansasAICampus**	**Bryson and Yao Team**
1	Peripheral oxygen saturation	The amount of time (in days) between each of the 12 most recent OMOP entries for a patient, and/or the number of entries available if that number is less than 12	Acute severe refractory exacerbation of asthma
2	Emergency Room Visit	Race	Edema, generalized
3	Drug test(s), presumptive, any number of drug classes, any number of devices or procedures; by instrument chemistry analyzers (eg, utilizing immunoassay [eg, EIA, ELISA, EMIT, FPIA, IA, KIMS, RIA]), chromatography (eg, GC, HPLC), and mass spectrometry eit	Protein S actual/normal in Platelet poor plasma by Coagulation	Gestation period, 28 weeks
4	MCHC [Mass/volume] by Automated count	Collection duration of Urine	Late effect of medical and surgical care complication
5	Acute renal failure syndrome	Patient age	The location id of the person
6	Postoperative state	Prostate specific Ag [Mass/volume] in Serum or Plasma	Deficiency of macronutrients
7	Hematocrit [Volume Fraction] of Blood by Automated count	Other cells/100 leukocytes in Body fluid by Manual count	Psychoactive substance use disorder
8	Leukocytes [#/volume] in Blood by Automated count	Anion gap in Serum or Plasma	Disease of the respiratory system complicating pregnancy, childbirth and/or the puerperium
9	Oxygen [Partial pressure] in Venous blood	Specific gravity of Urine by Automated test strip	Coag./bleeding tests abnormal
10	Creatinine [Mass/volume] in Serum or Plasma	Lymphocytes/100 leukocytes in Bronchial specimen	Complication occurring during pregnancy

© 2021 Yan Y et al. *JAMA Network Open.*

eAppendix 5. Ensemble Model

Top models for each of the challenge questions (10 for Q1 and 7 for Q2) were retrained separately on cumulative training dataset. The 10 trained models were applied on cumulative evaluation dataset and ensemble validation dataset to generate prediction correspondingly - the individual model would output a confidence interval between 0 and 1 indicative of the likelihood of a patient test positive for COVID-19 (Q1) or hospitalized within 21 days after testing positive in outpatient settings (Q2). A logistic regression model with 10-fold cross validation was trained on individual predictions generated on the cumulative evaluation dataset. The trained ensemble model was then applied on the individual predictions generated on ensemble validation dataset to generate an ensemble prediction. AUROC and AUPRC were computed by comparing the ensemble prediction to the gold standard for ensemble validation dataset. (eFigure 3)

The Q1 ensemble model combining the top 10 teams reached an AUROC of 0.714 (95%CI 0.713-0.715) and AUPRC of 0.106, compared to Q1 best individual model's AUROC of 0.699 (95%CI 0.698-0.700) and AUPRC of 0.112. When stratifying the ensemble validation dataset based on demographics profile, Q1 ensemble model outperformed the best individual model in 10 of total 13 subgroups. ($P<.001$, eFigure4, eTable 13) The Q2 ensemble model combining the top 7 teams reached an AUROC of 0.740 (95%CI 0.739-0.742) and AUPRC of 0.286, compared to Q2 best individual model's AUROC of 0.772 (95%CI 0.771-0.774) and AUPRC of 0.193.

© 2021 Yan Y et al. *JAMA Network Open.*

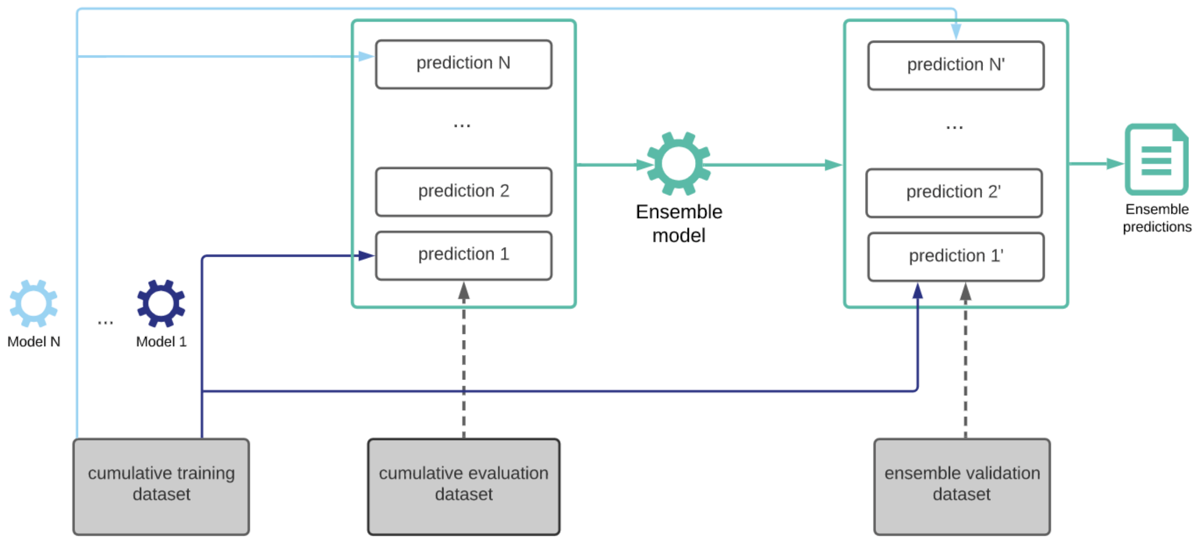

eFigure 3. Ensemble Model Diagram

© 2021 Yan Y et al. *JAMA Network Open.*

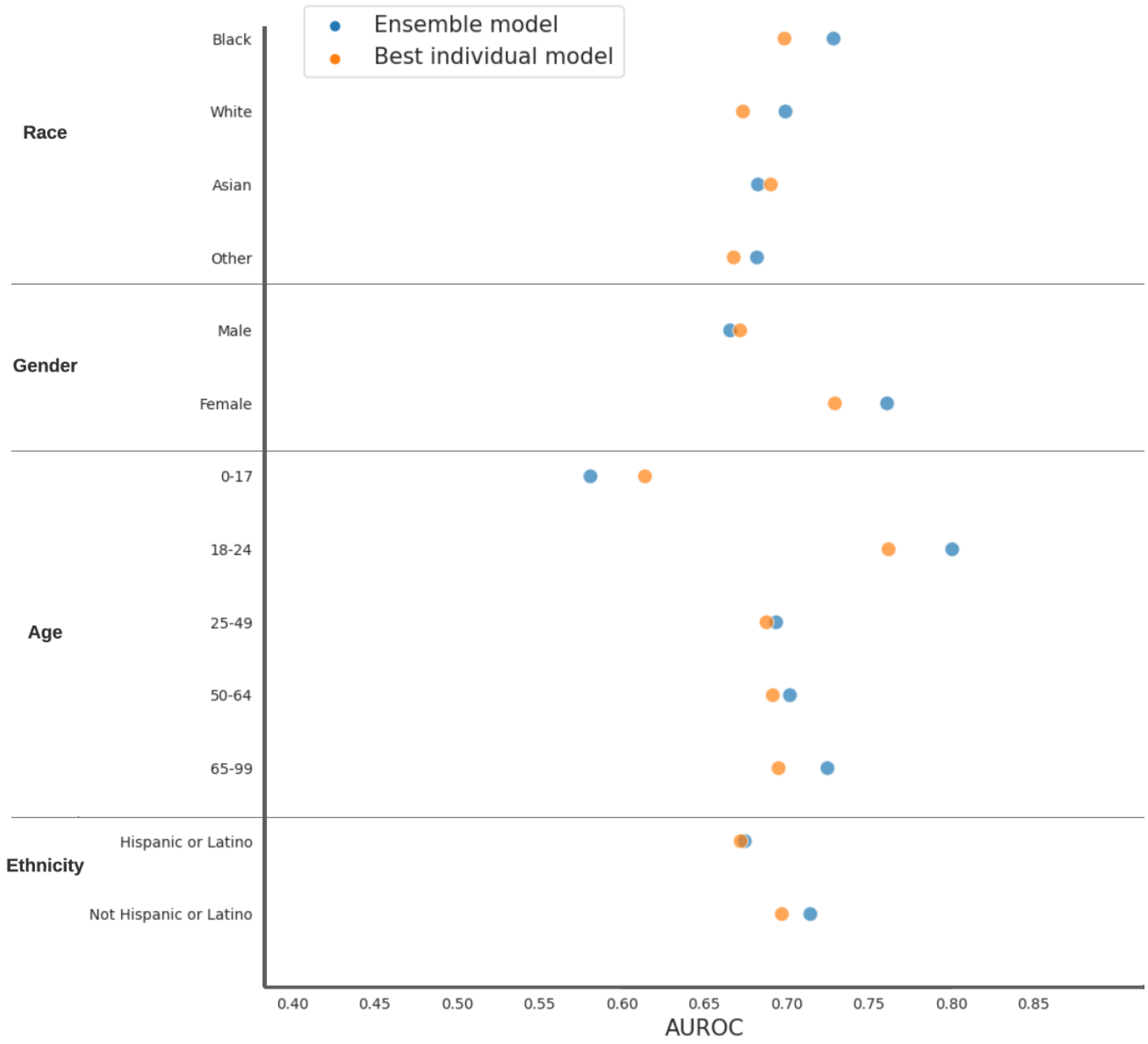

eFigure 4. Model Performance Comparison Between Question 1 Ensemble Model and Question 1 Best Individual Model on Demographics Subgroups Using Ensemble Validation Dataset

© 2021 Yan Y et al. *JAMA Network Open.*

eTable 13. Model Performance (AUROCs and 95% CI of AUROCs) Comparison Between Question 1 Ensemble Model and Question 1 Best Individual Model on Demographics Subgroups

		**Ensemble model**	**Best individual model**
**Race**	Black	0.729(0.728, 0.730)	0.699(0.698, 0.700)
	White	0.699(0.698, 0.700)	0.676(0.675, 0.677)
	Asian	0.683(0.682, 0.684)	0.689(0.688, 0.690)
	Other	0.682(0.681, 0.683)	0.668(0.667, 0.669)
**Gender**	Male	0.666(0.665, 0.667)	0.671(0.670, 0.672)
	Female	0.760(0.759, 0.761)	0.730(0.728, 0.731)
**Age**	0-17	0.581(0.580, 0.582)	0.614(0.613, 0.616)
	18-24	0.800(0.800, 0.801)	0.762(0.761, 0.763)
	25-49	0.694(0.693, 0.695)	0.687(0.686, 0.688)
	50-64	0.702(0.701, 0.703)	0.691(0.690, 0.693)
	65-99	0.724(0.723, 0.726)	0.696(0.695, 0.698)
**Ethnicity**	Hispanic or Latino	0.675(0.674, 0.676)	0.673(0.672, 0.674)
	Not Hispanic or Latino	0.714(0.713, 0.716)	0.696(0.695, 0.698)

© 2021 Yan Y et al. *JAMA Network Open.*

eAppendix 6. Top Teams' Model Description

Question 1 write-up

Home Sweet Home

Zafer Aydin[1], Amhar Jabeer[1]
[1]Department of Computer Engineering, Abdullah Gul University, Kayseri, Turkey
E-mail: zafer.aydin@agu.edu.tr, amhar.jabeer@agu.edu.tr

1. Introduction
The methods developed by team Home Sweet Home include missing value imputation, concept id ranking, feature extraction, a two-step feature selection, feature normalization, and classification. Information contained in measurement, condition, observation, drug exposure, device exposure, procedure, and visit tables is used to derive features. In addition, age, gender, race, and ethnicity information is also included. Concept ids are ranked with respect to number of occurrences among COVID-19 patients tested as positive, which enables to sort features with respect to their importance for the pandemic. Home Sweet Home has been the best performing team so far in question 1 of the COVID-19 DREAM Challenge on the last six dataset versions. The software is available at https://aguedutr-my.sharepoint.com/:f:/g/personal/zafer_aydin_agu_edu_tr/EnA2BRYcJdpPoAk71tJcZ1EBz5Ck0KgnDhmKnocW4UwtPA?e=UDM6Qc.

2. Methods

2.1 Missing value imputation
We performed the following missing value imputation steps on data tables. The missing values in regular date fields or start date fields of the tables are filled with January 1st, 1900, in end date fields are filled with January 1st, 2100 and those in value_as_number field of the measurement table are filled with 0.0.

2.2 Ranking concept ids
In each table of train data, we ranked concept ids with respect to their frequency of occurrence counts in positively labeled person_ids. In computing the frequencies, we eliminated duplicates that are caused by multiple entries for the same person. We only considered data that could be related to covid-19 pandemic in the USA. For this purpose, we eliminated data if measurement_date, condition_end_date, device_exposure_end_date, drug_exposure_end_date, procedure_date, and visit_date_fields are equal to a date on or before January 1st, 2020.

2.3 Feature extraction
In order to meet time quota restrictions of the challenge, we selected a maximum of the first 100 concept ids from each table of train set after the ranking process explained in Section 2.2. The same concept ids are also used to extract features for the test set in order to have equal number of features in both sets. Four types of features are extracted from the data tables: multi-instance learning based, count based, age, and one hot encoding based. These are explained in detail below.

2.3.1 Multi-instance learning features
In this work, for each measurement_concept_id selected from the measurement table, minimum, maximum, and average of the value_as_number fields are computed as multi-instance learning (MIL) features. For a given person_id and measurement_concept_id, if measurement data exists after January 1st, 2020 the MIL features are computed using data in this timeframe only. Otherwise, the MIL features are computed using data before this date if available. For the measurement_concept_id 3003694, which represents blood and Rh group, an ordinal encoding approach is used, which represents the feature values by integers from 0 to 7.

2.3.2 Count features
Count features are computed for measurement, condition, observation, drug exposure, device exposure, procedure, and visit tables. For instance, the measurement count feature includes the number of times a given measurement is present in the measurement table for a given person_id and measurement_concept_id. For drug exposure, drug quantity information is used as the count value. For the rest of the tables, a count of 1 is used for each entry. No time window constraint is applied for deriving the observation counts. For the remaining tables, count data after January 1st, 2020 is used only.

2.3.3 Age and one-hot encoding features

The age of each person is computed from the year of birth data available in person.csv file and is used as a single numeric feature. Gender, race, and ethnicity features are represented using a one-hot encoding approach applied to each feature separately.

2.4 Feature selection step 1

A wrapper-based feature selection strategy is employed on each data matrix of the train set separately (excluding age, gender, race, and ethnicity). A forward selection approach is used based on the ranking information derived as in Section 2.2. Starting from the empty set, in each iteration, a feature is included to the feature set and a 2-fold cross-validation is performed on the data matrix using lightGBM as the classifier [1] and stratified sampling to assign data samples to folds. The optimum feature set is found as the one that maximizes the AUPRC score. For MIL-based measurement features, once a feature is selected during the forward search, it is selected simultaneously from the three data matrices that contain minimum, maximum, and average values. The optimum number of features found for each table of the train set are used to select features for the tables of the test set directly using the same ranking information obtained for the train set.

2.5 Feature selection step 2

Data matrices are concatenated along the feature dimension (excluding gender, race, and ethnicity matrices). A second feature selection is performed on this concatenated matrix, which uses an embedded selection strategy. For this purpose, SelectFromModel module [2] of the scikit-learn library [3] of Python is employed, in which the base learner is set to lightGBM [1] with default settings. The same features selected for the train set are also selected for the test set.

2.6 Feature normalization

The data matrices excluding the matrices for gender, race, and ethnicity are normalized to the interval [0,1] using min-max scaling strategy [4].

2.7 Classification

The data matrix obtained at the end of the second feature selection step is concatenated with the data matrices for gender, race and ethnicity. In the training phase, a lightGBM classifier [1] with default settings is trained on this dataset and its learned parameters are used to compute predictions for the evaluation phase.

3. Results

Table 1 shows the leaderboard results of the team Home Sweet Home for four dataset versions of question 1. The team has been ranked as first on these datasets.

Table 1: Results of Home Sweet Home for challenge question 1

AUPRC	0.1154	0.2664	0.3102	0.4069
AUROC	0.7211	0.8095	0.8243	0.8222
Dataset	Week 26-30	Week 22-25	Week 18-21	Week 14-17

4. References

[1] lightGBM: https://lightgbm.readthedocs.io/en/latest/
[2] SelectFromModel: https://scikit-learn.org/stable/modules/generated/sklearn.feature_selection.SelectFromModel.html
[3] scikit-learn: https://scikit-learn.org/stable/
[4] Min-max scaler: https://scikit-learn.org/stable/modules/generated/sklearn.preprocessing.MinMaxScaler.html

© 2021 Yan Y et al. *JAMA Network Open.*

UWisc-Madison-BMI

Jifan Gao[1,2], Guanhua Chen[1,2]

[1] Department of Biostatistics and Medical Informatics, University of Wisconsin-Madison, Madison, Wisconsin, USA

[2] Contact: J.G. (jifan.gao@wisc.edu), G.C. (gchen25@wisc.edu)

Introduction

The task of Question 1 is to predict the risk that a patient's first SARS-CoV-2-test is positive given the patient's past EHR. We process diagnosis and measurement information and build a LightGBM model to make the prediction. Features are selected based on recent epidemiological studies [1,2].

Methods

We build binary indicators to show whether the following conditions and measurement exist after February 1st, 2020. 29 condition features and 23 measurement features are selected, we include the concept id and concept name of each feature we have selected: '254761': Cough, '259153': Pain in throat, '378253': Headache, '437663': Fever, '4185711': Loss of sense of smell, '43530714': Sensory disorder of smell and/or taste, '255848': Pneumonia, '256722': Bronchopneumonia, '256723': Pneumonia and influenza, '261326': Viral pneumonia,'443410': Infective pneumonia, '437677': Abnormal findings on diagnostic imaging of lung, '4305080': Abnormal breathing, '4223659': Fatigue, '442752': Muscle pain, '4195085':Nasal congestion, '45757468': Vomiting without nausea, '27674': Nausea and vomiting, '31967': Nausea, '441408': Vomitting, '196523': Diarrhea, '80141': Functional diarrhea, '4057826': Irritable bowel syndrome with diarrhea, '4168213': Chest pain on breathing, '372448': Loss of consciousness, '380844': Prolonged loss of consciousness, '381135': Brief loss of consciousness, '435786': Disorder of sleep-wake cycle, '436522': Irregular sleep-wake pattern,'3020891': Temperature, '3027018': Heart rate, '3012888': Diastolic blood pressure, '3004249': Systolic blood pressure, '3023314': Hematocrit, '3013650': Neutrophils, '3004327': Lymphocytes, '3016502': SpO2, '4196147': Peripheral oxygen saturation, '3044938': Influenza virus A RNA [Presence] in Unspecified specimen by NAA with probe detection, '3044254': Respiratory syncytial virus RNA [Presence] in Unspecified specimen by NAA with probe detection, '3042596': Human coronavirus RNA [Presence] in Unspecified specimen by NAA with probe detection, '3042194': Human metapneumovirus RNA [Presence] in Unspecified specimen by NAA with probe detection, '3038297': Parainfluenza virus 4 RNA [Presence] in Unspecified specimen by NAA with probe detection, '3016723': Creatinine [Mass/volume] in Serum or Plasma, '3023103': Potassium [Moles/volume] in Serum or Plasma, '3025634': Parainfluenza virus 1 RNA [Presence] in Unspecified specimen by NAA with probe detection, '3000963': Hemoglobin [Mass/volume] in Blood, '3000905': Leukocytes [#/volume] in Blood by Automated count, '3008037': Lactate [Moles/volume] in Venous blood, '3005491': Lactate [Moles/volume] in Plasma venous, '3022250': Lactate dehydrogenase [Enzymatic activity/volume] in Serum or Plasma by Lactate to pyruvate reaction, '706181': SARS coronavirus 2 IgG Ab [Presence] in Serum or Plasma by Immunoassay.

Combining demographics information (gender, age, and race) and features listed above, there are 55 features in total for each patient. A LightGBM model is used to make the prediction. Missing values (if any) in the measurement data are handled by the LightGBM: the missing values are ignored when the model decides where to split and then are allocated to the branch where the loss is reduced the most [3]. Some of the hyperparameters are set as follows and others remain default values:'objective': 'binary';'metric': 'cross_entropy';'learning_rate': 0.01;'num_leaves': 32;'feature_fraction': 0.95.

Results

The AUROC and AUPR are 0.738 and 0.1127 respectively with the dataset version of "Week 18-21".

Reference

[1] Sun Y, Koh V, Marimuthu K, Ng OT, Young B, Vasoo S, Chan M, Lee VJ, De PP, Barkham T, Lin RT. Epidemiological and clinical predictors of COVID-19. Clinical Infectious Diseases. 2020 Jul 28;71(15):786-92.

[2] Siordia Jr JA. Epidemiology and clinical features of COVID-19: A review of current literature. Journal of Clinical Virology. 2020 Apr 10:104357.

[3] "What Happens With Missing Values During Prediction? · Issue #2921 · Microsoft/Lightgbm". *Github*, 2021, https://github.com/microsoft/LightGBM/issues/2921.

© 2021 Yan Y et al. *JAMA Network Open.*

Bryson and Yao Team

Yuxin Yao[1], Kevin Bryson[2]
Department of Computer Science, University College London, London, UK
Email:[1] yuxin.yao.19@ucl.ac.uk, [2] k.bryson@ucl.ac.uk

Introduction

This model is based on logistic regression and developed to predict if a patient will test positive for COVID-19 using the scikit-learn package [1]. The model is developed in order to obtain high accuracy and true positive rate.

Method

The age, ethnicity, gender and race of patients, together with nine conditions and four measurements are selected as features of our model. The conditions are dyspnea, cough, fever, sore throat, headache, pneumonia, muscle pain, loss of smell and fatigue. These are the common conditions observed on COVID-19 patients and they have a high correlation with COVID-19 [2]. The measurements are diastolic blood pressure, C-reactive protein measurement, lymphocyte count, and lactate dehydrogenase measurement [2,3]. All of these features were put into the logistic model as binary features (values 0.0 or 1.0), with the measured values binarized by comparing them with the normal standard value for that measurement.

According to Johns Hopkins Coronavirus Resource Center [4], the rate of positivity of Covid-19 tests varies between 5% and 20% over the past year, implying the database would contain a majority of negative results whose proportion may vary over time. This unbalanced data can lead to a biased model that predicts lots of positive cases negative. This problem was avoided by using a weighted logistic regression model that automatically adjusts weights inversely proportional to class frequencies in the input data.

To facilitate generalization and avoid over-fitting for what are initially fairly small datasets, we employed elastic net regularization, using a grid-search cross-validation strategy to determine optimal L1 and L2 regularization parameters, evaluated using the F1 score.

Result Discussion

The models are tested by Dream Challenge with the data set version "Week 12-13" and the AUROC and AUPRC are given as feedback.

With the simplest logistic regression model that trained with the features selected above, the AUROC is 0.6566 and AUPRC is 0.0793. With the weight logistic regression model, the AUROC increased to 0.6604 and AUPRC to 0.0817. After applying elastic net regularization and grid search, the AUROC and AUPRC increased significantly to 0.7375 and 0.0979 respectively, resulting in a significant increase in true positive rate.

Reference

[1] https://scikit-learn.org/

[2] Feng C, Huang Z, Wang L, Chen X, Zhai Y, Zhu F, et al.A NovelTriage Tool of Artificial Intelligence Assisted Diagnosis Aid System for Suspected COVID-19 pneumonia In Fever Clinics. medRxiv. 2020. Available from:https://www.medrxiv.org/content/early/2020/03/20/2020.03.19.20039099.

[3] Henry BM, Aggarwal G, Wong J, Benoit S, Vikse J, Plebani M, et al. Lactate dehydrogenase levels predict coronavirus disease 2019 (COVID-19) severity and mortality: A pooled analysis. The American journal of emergency medicine. 2020 Sep;38(9):1722–1726. 32738466[pmid]. Available from: https://pubmed.ncbi.nlm.nih.gov/32738466.

[4] Johns Hopkins Coronavirus Resource Center . Daily state-by-state testing trends. Available from: https://coronavirus.jhu.edu/testing/individual-states

© 2021 Yan Y et al. *JAMA Network Open.*

Question 2 write up

Ivanbrugere

Ivan Brugere[1], Lav R. Varshney[2]
[1]Department of Computer Science, University of Illinois at Chicago
[2]Department of Electrical and Computer Engineering, University of Illinois Urbana-Champaign
E-mail: ivan@ivanbrugere.com, varshney@illinois.edu

Introduction
Our proposed method for Question 2 of the EHR Dream Challenge - COVID-19 prediction focuses on diverse model selection with the fewest domain assumptions. We find that gradient-boosted trees are competitive against more sophisticated embedding-based methods and achieve the highest performance on Q2 without consideration of data date ranges, imputation, or other feature engineering.

Method
Our method does hyperparameter grid search over gradient-boosted tree models AdaBoost [1] and Catboost [2]. We select the model with maximum mean AUROC over (k=3) model instantiations using random training/validation partitions (p=0.5) of input training data. All models are evaluated over the same training/validation samples (e.g. there are exactly k=3 partitions over the model selection).

Data
We build a binary feature vector over all concepts, over all time within the patient history. Within the "person" table, we drop "day_of_birth" and "time_of_birth" fields and otherwise create a one-hot encoding of categorical values, including "year_of_birth."
We apply this one-hot encoding over all concepts in all tables, yielding 6209 total binary features.

Hyperparameters
We use the following hyperparameter grid search for each method:

AdaBoost	Catboost
Depth: 5<br>N: 200<br>Learning rate: 0.1, 0.25	Depth: 5<br>Objectives: "Logloss", "CrossEntropy"<br>N: 100, 200<br>Learning rate: 0.01, 0.05, 0.1, 0.5<br>L2 leaf regularization: 1, 5, 9,13<br>Features (rsm): 0.5, 0.75<br>Class weights: "Balanced", Null

In the above 'N' denotes the number of ensemble trees, random subspace method (rsm) denotes the proportion of features to be randomly sampled per split within the tree.
The final model selected is:

Catboost
Depth: 5<br>Objective: "Logloss"<br>N: 200<br>Learning rate: 0.01<br>L2 leaf regularization: 13<br>Features (rsm): 0.50<br>Class weights: "Balanced"

© 2021 Yan Y et al. *JAMA Network Open.*

As expected, model selection chooses a larger ensemble (N: 200) and does class balancing. Finally, the model uses the largest weight regularization (L2 leaf regularization: 13) to avoid overfitting.

Analysis

This methodology allows for novel competing models to be quickly added in model selection without loss of model performance (e.g. the prior best model may still be selected). For example, we included neural network embedding methods within the model selection but did not see improved performance. We had limited visibility to the model selected on real data, so we could not confirm that a gradient-boosting model was chosen. However, we found better improvements in wider gradient-boosting hyperparameter search and more robust train/validation sampling at increased k. Without compute budgeting, model selection over all of these would strictly improve performance vs. higher compute cost.

We did not consider any temporal windowing to train only on "recent" visits. We started with the simplest model and found it surprisingly competitive without these filters. It is likely that these predictive features are rare within the entirety of the patient record. The learned association may not incur Type I errors due to prior events not related to recent covid treatments.

Reference

[1] https://scikit-learn.org/

[2] https://catboost.ai/

Arkansas AI Campus (ArkansasAICampus20)

Jason L. Causey[1,2] on behalf of the ArkansasAICampus20 team.

[1] Computer Science Department, College of Engineering and Computer Science, Arkansas State University

[2] Arkansas AI-Campus, Center for No-Boundary Thinking, Arkansas State University

jcausey@astate.edu

Model Description

An important aspect of this challenge was that participating teams were unable to see real patient data. The simulated data provided for model testing provided little information about which features might correlate to patient hospitalization risk, so we built a flexible framework for evaluating alternative models and automated feature engineering. We chose the ExtraTreesClassifier from the Scikit-Learn Python library (https://scikit-learn.org) based on empirical performance. Other models we considered included XGBoost1 and LightGBM.2

Training: Our model was trained by first performing the preprocessing feature extraction described below, then simple random oversampling of the existing positive examples was used to achieve an overall positive ratio of 40%. This augmented dataset was used to train ExtraTreesClassifier, with the following parameters: 'n_estimators': 1800, 'max_depth': 4, 'max_features': 'sqrt', 'bootstrap': True, 'n_jobs': -1, 'oob_score': True, 'class_weight': 'balanced_subsample', 'random_state': 0.

A secondary logistic regression model (LogisticRegression from Scikit-Learn) was fit to the predictions produced by ExtraTreesClassifier. Its presence provided a consensus model for ensembles; it was active in this version even though only one ExtraTreesClassifier was employed.

Data Preprocessing

We utilized information from the person, measurement, observation, condition_occurrence, and drug_exposure tables, as well as dates of contact from the procedure_occurrence, device_exposure, and visit_occurrence tables to determine the last recorded date of contact with each patient.

Features from the person table were race (5 features), ethnicity (2 features,), gender (2 features), and an engineered age feature derived from the difference between a patient's date of birth and last contact date. We used 38 raw features from the condition_occurrence table, selected manually. We used 11 raw features from the drug_exposure table, all hand selected. 207 raw features from the measurement table were used, selected by a combination of manual choice and random selection. The observation table provided four hand selected features: "Cardiac rhythm", "Blood pressure method", "Tobacco user", "History of alcohol use".

After the raw features were selected, we performed an automated feature engineering / expansion process. A Boolean feature valid_test_flag was added indicating whether the patient had an entry for a COVID-19 test within 21 days of the last contact date. For each raw feature, we derived and added the following: A Boolean indicating whether the value was present or missing, the standardized feature (=0, =1), the standard deviation, a numeric measurement of the

© 2021 Yan Y et al. *JAMA Network Open.*

amount of missing data. We reduced the size of the dataset, retaining the most recent 12 values for each feature, a "baseline" feature storing the oldest entry for each feature, and a numeric indicator of where in the dataset the baseline value was found. After the feature expansion, our dataset contained 15,232 features, named according to the following pattern:
FEATURENAME_tX: (X [0,11]), the raw (12−t) th newest measurement recorded for FEATURENAME.
FEATURENAME_notna: flag indicating value of FEATURENAME was not missing
FEATURENAME_normed_tX: (X [0,11]), the standardized (=0, =1) (12−t) th newest measurement recorded for FEATURENAME.
FEATURENAME_std: standard deviation for this feature
FEATURENAME_sprc: a measure of sparsity for this feature
FEATURENAME_bl: oldest available value for FEATURENAME
FEATURENAME_tdelta: analog for relative age of FEATURENAME_bl (larger value implies older)
FEATURENAME is derived from the OMOP table name and concept id, e.g., "measurement_3000067" (measurement table, concept ID 3000067).
Engineered features not directly from the OMOP tables are:
age: an analog for patient age, calculated as the difference between last contact date and birth date.
delta: measures the time difference (in negative days) from the last known contact for that patient for each feature.
valid_test_flag: Boolean indicating whether the patient had a record of a COVID-19 test less than 22 days from the last contact date.
We define "contact" to mean any dated entry in any OMOP table attributed to that patient ID. "Last contact" is the most recent contact for the corresponding patient.
The automated feature expansion process produces redundant features for values "age", "gender", "race", "ethnicity", and "delta" since those values cannot re-occur, but are still expanded into "timestep" and "baseline" columns), and some are Boolean in nature, but a "normed" version and standard deviation computed and added to the dataset. For this reason, features such as race_8552_t8 and race_8552_t9 contain identical information and should be viewed as a single feature. Any of these might be selected to play a role in the underlying decision trees.

References
1. Chen, T. & Guestrin, C. XGBoost: A Scalable Tree Boosting System. Proceedings of the 22nd ACM SIGKDD International Conference on Knowledge Discovery and Data Mining - KDD '16 785–794 (2016). doi:10.1145/2939672.2939785
2. Ke, G. et al. LightGBM: A highly efficient gradient boosting decision tree. in Proceedings of the 31st International Conference on Neural Information Processing Systems 3149–3157 (Curran Associates Inc., 2017).

© 2021 Yan Y et al. *JAMA Network Open.*

Bryson-and-Yao-Team

Yuxin Yao[1], Kevin Bryson[2]
Department of Computer Science, University College London, London, UK
Email:[1] yuxin.yao.19@ucl.ac.uk, [2] k.bryson@ucl.ac.uk

Introduction

This model was based on logistic regression and developed to predict if a patient who has tested positive for COVID-19 in out-patients will be hospitalized within 21 days of their COVID-19 test.

Method

Logistic regression,implemented within scikit-learn [1], was used to develop the model. The features are selected from patients' information, condition occurrences and measurements. All the condition occurrences that appeared after 2015 are extracted. The four hand-selected measurements that appeared after 2019 are extracted. The measurements are diastolic blood pressure, C-reactive protein measurement, lymphocyte count, and lactate dehydrogenase measurement [1,2]. These are turned into binary features by comparing each measurement to its normal expected standard range. Besides the patients' information like gender, ethnicity, race and age, the location id which indicates the address of patients is included in the features before feature selection, because the numbers of positive cases are different among areas, and the hospitality rates are different, which may influence the prediction.

Features are selected by using the chi-square value, where the chi-square values of all the features in the training set are calculated and ranked. The calculation is fast and easy, so it is helpful to select features. The best fifty features with the best chi-square value was selected to give the features used in model training. The hospitality rate for different areas are not published, so it is hard to know if the dataset is unbalanced. Thus, we used a weighted logistic regression model so that the weight of input data is adjusted, to eliminate any bias if the data is unbalanced. Elastic net regularization with grid search on the ratio between L1 and L2 regularization is employed to achieve the best model. The F1 score acts as the evaluation of models created during the grid search using cross-validation. The model with the greatest F1 score is then finally selected.

Result

The models were tested by Dream Challenge with the data set version "Week 1-4", and it achieved AUROC: 0.6696 and AUPRC: 0.1714.

Reference

[1] https://scikit-learn.org/

[2] Feng C, Huang Z, Wang L, Chen X, Zhai Y, Zhu F, et al.A NovelTriage Tool of Artificial Intelligence Assisted Diagnosis Aid System for Suspected COVID-19 pneumonia In Fever Clinics. medRxiv. 2020. Available from:https://www.medrxiv.org/content/early/2020/03/20/2020.03.19.20039099.

[3] Henry BM, Aggarwal G, Wong J, Benoit S, Vikse J, Plebani M, et al. Lactate dehydrogenase levels predict coronavirus disease 2019 (COVID-19) severity and mortality: A pooled analysis. The American journal of emergency medicine. 2020 Sep;38(9):1722–1726. 32738466[pmid]. Available from: https://pubmed.ncbi.nlm.nih.gov/32738466

© 2021 Yan Y et al. *JAMA Network Open.*
