# Supplementary material for: A Continuously Benchmarked and Crowdsourced Challenge for Rapid Development and Evaluation of Models to Predict COVID-19 Diagnosis and Hospitalization
Source: JAMA Netw Open. 2021 Oct 11;4(10):e2124946. doi: 10.1001/jamanetworkopen.2021.24946 (PMC8506231; doi:10.1001/jamanetworkopen.2021.24946)
Supplement: Supplement 2. — Nonauthor Collarborators. DREAM Challenge Consortium [file jamanetwopen-e2124946-s002.pdf]

Supplemental Online Content: Nonauthor Collaborators

*Indicates required information. Only first name, last name, and suffix will appear in PubMed.

***Group Name(s):** the DREAM Challenge Consortium							
***First Name and Middle Initial(s)**	***Last Name**	***Suffix (eg, Jr, III)**	Academic Degrees	Institution	Location (city, state/province, country)	Role or Contribution, eg, chair, principal investigator	Group (if more than 1 Group listed in the byline) and/or Subgroup (eg, Steering Committee)
Chethan	Jujjavarapu		BS	Biomedical Informatics and Medical Education, University of Washington	Seattle, WA, USA		
jason	thomas		BS	University of Washington Department of Biomedical Informatics and Medical Information	Seattle, WA, USA		
Martin	Gunn		MBChB	Department of Radiology, University of Washington	Seattle, WA, USA		
YiFan	Wu		MPH	Biomedical and Health Informatics, University of Washington	Seattle, WA, USA		
Nicholas	Dobbins		MLIS	Department of Biomedical Informatics & Medical Education, University of Washington ; Research Information Technology University of Washington Medicine	Seattle, WA, USA		
Vikas	O'Reilly-Shah		MD, PhD, FASA	Department of Anesthesiology & Pain Medicine, University of Washington; Department of Anesthesiology & Pain Medicine, Seattle Children's Hospital	Seattle, WA, USA		
Andrew	Teng		MS	Biomedical Informatics and Medical Education, University of Washington	Seattle, WA, USA		
Noah	Hammarlund		PhD	School of Pharmacy, The Comparative Health Outcomes, Policy, and Economics (CHOICE) Institute, University of Washington; Biomedical Informatics and Medical Education, University of Washington	Seattle, WA, USA		
Graham	Nichol		MD MPH	University of Washington-Harborview Center for Prehospital Emergency Care; Departments of Medicine and Emergency Medicine, University of Washington	Seattle, WA, USA		
Pascal	Brandt		MSc	Biomedical Informatics and Medical Education, University of Washington	Seattle, WA, USA		
Vikas	Pejaver		PhD	[illegible] Washington	Seattle, WA, USA		
Beth	Britt		PhD	ITS Analytics, UW Medicine	Seattle, WA, USA		
Yuanfang	Guan		PhD	Department of Computational Medicine and BIoinformatics, University of Michigan	Ann Arbor, MI, USA		
Lingrui	Cai		MSc	Ann Arbor Algorithms	Ann Arbor, MI, USA		
Kaiman	Zeng		PhD	Department of Electrical Engineering, College of Engineering and Applied Sciences, Arkansas Tech University	Russellville, Arkansas, USA		
Bruce	Cragin		PhD	Wind City Applied Research	Lempster, NH, United States		
Shirya	Kaul		MSc	Science and Engineering faculty , Queensland university of Technology	Brisbane, Queensland, Australia		
Jennifer	Fowler		BS	Arkansas State University, Arkansas AI Campus	Jonesboro, AR, USA		
Oznur	Tastan		PhD	Faculty of Science and Engineering, Sabanci University	Tuzla, İstanbul, Turkey		
Vladimir	Kovacevic		PhD	Department for Computer science, School of Electrical engineering, University in Belgrade	Belgrade, Serbia		
Ege	Alpay		BS	Graduate School of Engineering and Natural Sciences, Sabanci University	Istanbul, Turkey		
Luiza	Romanovskii-Chernik		MSc	Department of Software and Information Systems Engineering, Ben-Gurion University of the Negev	Beer Sheva, Israel		
Aleksandr	Romanovskii-Chernik		MSc	Department of Software and Information Systems Engineering, Ben-Gurion University of the Negev	Beer Sheva, Israel		
Alper	Bingol		-	Computer Science and Engineering, Faculty of Engineering and Natural Science, Sabanci University	Istanbul, Turkey		
Sema	Yılmazer		-	Computer Science and Engineering, Faculty of Engineering and Natural Science, Sabanci University	Istanbul, Turkey		
Shankai	Yan		PhD	National Center for Biotechnology Information, National Library of Medicine, National Institutes of Health	Bethesda, Maryland, USA		
Santina	Lin		MSc	College of Computing, Georgia Institute of Technology	Atlanta, Georgia, USA		
Ege	Arıkan		BS	Computer Science and Engineering, Faculty of Engineering and Natural Science, Sabanci University	Istanbul, Turkey		
Lav	Varshney		PhD	Salesforce Research; University of Illinois at Urbana-Champaign	Urbana, IL, USA		
Jimmy	Phuong		PhD	Division of Biomedical and Health Informatics, University of Washington; UW Medicine Research Information Technology, University of Washington; Harborview Injury Prevention Research Center, University of Washington,	Seattle, WA, USA		
